# Aqueous Flow Reactor and Vapour‐Assisted Synthesis of Aluminium Dicarboxylate Metal–Organic Frameworks with Tuneable Water Sorption Properties†

**DOI:** 10.1002/chem.202001661

**Published:** 2020-07-27

**Authors:** Timothée Stassin, Steve Waitschat, Niclas Heidenreich, Helge Reinsch, Finn Pluschkell, Dmitry Kravchenko, João Marreiros, Ivo Stassen, Jonas van Dinter, Rhea Verbeke, Marcel Dickmann, Werner Egger, Ivo Vankelecom, Dirk De Vos, Rob Ameloot, Norbert Stock

**Affiliations:** ^1^ Centre for Membrane Separations, Adsorption, Catalysis and Spectroscopy for Sustainable Solutions (cMACS) KU Leuven Celestijnenlaan 200F box 2454 3001 Leuven Belgium; ^2^ Institut für Anorganische Chemie Christian-Albrechts-Universität zu Kiel Max-Eyth-Straße 2 24118 Kiel Germany; ^3^ Heinz Maier-Leibnitz Zentrum (MLZ) and Physik Department E21 Technische Universität München Lichtenbergstraße 1 85748 Garching Germany; ^4^ Institut für Angewandte Physik und Messtechnik LRT2 Universität der Bundeswehr München Werner-Heisenberg-Weg 39 85577 Neubiberg Germany

**Keywords:** flow reactors, metal–organic frameworks, tuneable properties, vapour-assisted synthesis, water adsorption

## Abstract

Energy‐efficient indoors temperature and humidity control can be realised by using the reversible adsorption and desorption of water in porous materials. Stable microporous aluminium‐based metal–organic frameworks (MOFs) present promising water sorption properties for this goal. The development of synthesis routes that make use of available and affordable building blocks and avoid the use of organic solvents is crucial to advance this field. In this work, two scalable synthesis routes under mild reaction conditions were developed for aluminium‐based MOFs: (1) in aqueous solutions using a continuous‐flow reactor and (2) through the vapour‐assisted conversion of solid precursors. Fumaric acid, its methylated analogue mesaconic acid, as well as mixtures of the two were used as linkers to obtain polymorph materials with tuneable water sorption properties. The synthesis conditions determine the crystal structure and either the MIL‐53 or MIL‐68 type structure with square‐grid or kagome‐grid topology, respectively, is formed. Fine‐tuning resulted in new MOF materials thus far inaccessible through conventional synthesis routes. Furthermore, by varying the linker ratio, the water sorption properties can be continuously adjusted while retaining the sigmoidal isotherm shape advantageous for heat transformation and room climatisation applications.

## Graphical abstract

**Going with the flow**: A collection of microporous aluminium fumarate‐mesaconate metal–organic frameworks (MOFs) are prepared by using an aqueous flow reactor and vapour‐assisted synthesis. The materials have a variable and, for some, previously unreported crystal structure, and their tuneable water sorption properties depend on the preparation method and linker content.

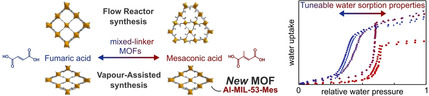

## Introduction

The broad family of porous materials finds widespread use in catalysis,^1^ adsorptive separations^2^ and ion exchange,^3^ among many other applications. As a relatively young branch of this family tree,^4^ metal–organic frameworks (MOFs) are under evaluation for several real‐life applications. They represent a versatile group of compounds with record‐breaking surface areas (>7000 m^2^ g^−1^)^5^ and promising properties for sensing,^6^ gas capture and separation^7^, ^8^ and heat exchange.^9^ Nevertheless, no large‐scale applications have been implemented thus far. One challenge lies in the often hazardous and low‐yielding synthesis conditions of MOFs. To overcome these challenges, synthesis protocols suitable for industrial scale‐up have to be developed while taking into account pricing of the final product.^10^, ^11^ In this respect, a particularly interesting MOF is aluminium fumarate, also known as Al‐MIL‐53‐Fum (MIL=Material Institute Lavoisier).^12^ It exhibits high porosity and stability, even under hydrothermal stress,^13^ and therefore has sparked the interest of industrial researchers.^14^ In particular, the sigmoidal water ad‐/desorption curve without hysteresis demonstrated by Al‐MIL‐53‐Fum and other Al‐MOFs is attractive for applications in heat‐exchange devices.^15^, ^16^, ^17^, ^18^, ^19^ The patented synthesis of Al‐MIL‐53‐Fum is a hydrothermal batch process that makes use of inexpensive and readily available starting materials (fumaric acid, NaOH, aluminium sulfate) and avoids hazardous solvents such as dimethylformamide.^20^ Similar, mild synthesis conditions (≤100 °C) have been demonstrated for other Al‐MOFs,^21^, ^22^, ^23^, ^24^ thereby avoiding pressure build‐up. Also, synthesis methods based on extrusion, microwave‐assisted heating and starting from insoluble metal ion sources have been reported.^25^, ^26^, ^27^ For the further scale‐up of these MOFs, it can be desirable to move to continuous production in flow reactors as multiplying the output volume through multiple tubes in parallel enables more control compared with larger dimension batch reactors.^28^, ^29^ An alternative elegant approach to green and scalable MOF synthesis would be the conversion of non‐salt precursors using no or minimal amounts of solvent. Several oxide‐based solvent‐free syntheses or vapour‐assisted methods have been reported, although only for MOFs based on divalent metal ions (e.g., Cu^2+^, Zn^2+^).^30^, ^31^, ^32^ To date, vapour‐assisted synthesis of MOFs based on tri‐ and tetravalent metal ions (e.g., Fe^3+^, Al^3+^, Zr^4+^) has required the use of metal salts.^33^

**Figure 1:**
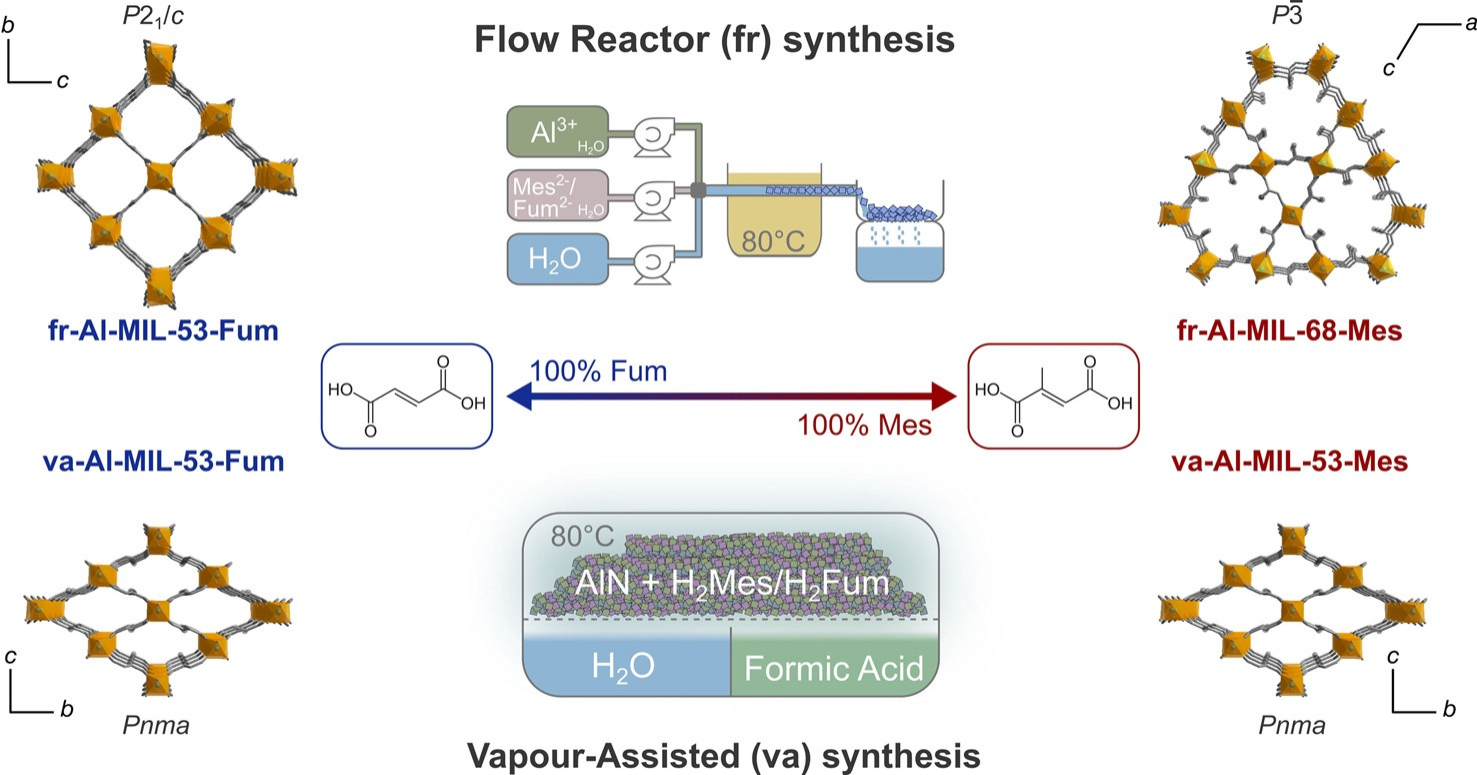
Flow reactor (fr) and vapour‐assisted (va) synthesis of MOFs. Starting from different aluminium precursors, but making use of the same fumaric and mesaconic acid linkers, single‐ and mixed‐linker frameworks can be obtained, with the topology and space group depending on the synthesis method.

In this contribution, we investigate the flow reactor (fr) and vapour‐assisted (va) synthesis of Al‐MIL‐53‐Fum and related mixed‐linker Al‐MOFs (Figure 1) to find efficient scalable preparation methods for materials with improved water sorption properties. Salt and non‐salt aluminium precursors were used. The tested linker molecules are fumaric acid (H_2_Fum) and mesaconic acid (methylfumaric acid, H_2_Mes), and combinations thereof. Although H_2_Fum is industrially available, H_2_Mes can be derived from readily available citric acid.^34^ The resulting MOFs crystallise either in a square‐ or kagome‐grid topology as observed in Al‐MIL‐53‐Fum^35^ and Al‐MIL‐68‐Mes,^35^ respectively. All materials were characterised for their structural and sorption properties.

## Experimental Section

### Flow reactor set‐up

The flow reactor set‐up is similar to the one recently described by the Stock group. Details regarding the reactor volume, flow rates and achievable temperatures are given in the Supporting Information (Section S2.1 in the Supporting Information).^36^, ^37^ The three syringes of the reactor are loaded with (i) an aqueous 0.05 m aluminium sulfate solution, (ii) an aqueous solution of linker mixtures (0.1 m) and KOH (0.3 m) and (iii) water, respectively (Figure 1, top). In a typical procedure, only the precursor solutions (i and ii) are initially pumped through the tubes and mixed via a quadruple cross connector before passing to the reactor, which consists of a coiled Teflon tube heated in an oil bath. Once the reactor is filled, the water syringe (iii) is used to push the remaining precursor solution and the product slurry out of the reactor. The obtained product was centrifuged and washed with ethanol.

### Vapour‐assisted conversion process

Mixtures of aluminium nitride (AlN) and linker powders (1:2 ratio, 200 mg) were placed in a 25 mL sealed glass bottle together with glass vials containing 1 mL of liquid to generate vapours (Figure 1, bottom). After 48 h reaction at 80 °C in a pre‐heated oven, the vapour and excess ligand were removed by heating the powder in vacuum at 200 °C for 2 h. Eventually, the material was calcined at 300 °C for 12 h.

### Additional information

Materials and methods, syntheses optimization details, structure refinements and fits, characterisation data (^1^H NMR, elemental analysis, TGA, FTIR, SEM, N_2_ sorption, PALS), and linker vapour pressure data can be found in the Supporting information.

Deposition Number 1918969 contains the supplementary crystallographic data for this paper. These data are provided free of charge by the joint Cambridge Crystallographic Data Centre and Fachinformationszentrum Karlsruhe Access Structures service www.ccdc.cam.ac.uk/structures.

## Results and Discussion

### Flow reactor (fr) synthesis

#### Single‐linker fr‐Al‐MIL‐53‐Fum and fr‐Al‐MIL‐68‐Mes

For the synthesis optimization of fr‐Al‐MIL‐53‐Fum, different metal‐to‐linker ratios (2:1, 1:1, 1:2) and different reaction times, determined by the flow rate, were investigated at a reaction temperature of 80 °C. A 1:1 metal‐to‐linker ratio and 15 min residence time in the flow reactor were identified as the optimal reaction conditions for the synthesis of fr‐Al‐MIL‐53‐Fum (Table S2.1 in the Supporting Information). For the synthesis of fr‐Al‐MIL‐68‐Mes, identical conditions (1:1 ratio, 15 min) were found to be optimal (Table S2.2 in the Supporting Information). The phase purity of both MOFs was confirmed by PXRD (Le Bail fits Figures S4.1 and S4.2 in the Supporting Information), the composition was confirmed by ^1^H NMR spectroscopy (Section S5 in the Supporting Information), elemental analysis (Section S6 in the Supporting Information), thermogravimetric analysis (Section S7 in the Supporting Information), IR spectroscopy (Section S8 in the Supporting Information) and the morphology was investigated by electron microscopy (Section S9 in the Supporting Information).

### Vapour‐assisted (va) synthesis

#### Aluminium nitride as a reactive precursor

Under mild conditions (80 °C), aluminium oxide will not react to form Al‐MIL‐53‐Fum, even in the presence of water vapour, whereas aluminium nitride does (Table S3.1 in the Supporting Information). The higher reactivity of the nitride is due to the softer Al−N bonds, which are favourably replaced by the harder Al−O bonds formed with dicarboxylate linkers.^38^ At room temperature in moist air (80 % relative humidity, RH), complete hydrolysis of aluminium nitride to aluminium hydroxide can be achieved, but only after prolonged reaction times (>400 h for micron‐sized particles).^39^ The conversion to Al‐MOFs requires a high relative humidity (94 %), yet is incomplete when only water vapour is present (Figures S3.2 and S3.4 in the Supporting Information). Likely, the MOF forming at the surface of micron‐sized aluminium nitride particles hinders further conversion.^40^ Indeed, surface treatment of aluminium nitride with various acids that bind to Al^3+^ (e.g., phosphoric acid, acetic acid) is known to delay or prevent hydrolysis.^41^

#### Solvent‐free activation conditions

Powder X‐ray diffraction (PXRD) measured for the as‐synthesized materials reveals the presence of the MOF, excess ligand and unreacted aluminium nitride (Figure S3.4 in the Supporting Information). To avoid washing with organic solvents, a two‐step activation treatment was optimized that takes advantage of the high thermal stability of Al‐MIL‐53 materials^42^ (>350 °C): (1) sublimation of the excess linker at 200 °C under vacuum, followed by (2) calcination at 300 °C to remove adsorbed linker from the pores. Without calcination, no porosity is detected (Brunauer–Emmett–Teller, BET, surface area <25 m^2^ g^−1^), whereas after calcination the Type I isotherm expected for microporous materials is observed (Figure S3.3 in the Supporting Information). In situ temperature‐dependent PXRD shows the sublimation of crystalline linker around 200 °C. Also, the intensity of the framework reflection at approximately 13.3°, indicative of electron density in the pores, thus linker molecules, gradually disappears around 300 °C (Figures S3.6 and S3.7 in the Supporting Information). Still, calcination steps bring additional energetic costs and should therefore preferably be performed by using low‐grade waste heat, thus at temperatures ≤250 °C.^43^

#### Formic acid vapour as synthesis modulator

Formic acid has been used elsewhere to modulate the solvothermal synthesis of Al‐MIL‐53‐Fum, resulting in improved isotherms and kinetics for water adsorption.^44^ When applied to the vapour‐assisted synthesis of Al‐MIL‐53‐Fum, full conversion of aluminium nitride can be achieved through the addition of formic acid vapour to the reaction atmosphere. Moreover, the presence of formic acid vapour allows MOF formation under lower relative humidity (79 %; Figure S3.8 in the Supporting Information). Only a trace amount of nitrogen and formate ions (<1 %) is found in the final product by elemental analysis and ^1^H NMR spectroscopy, respectively (Sections S5 and S6 in the Supporting Information). Formate is likely incorporated in the framework during synthesis, but it is removed upon thermal activation.^45^ The organic content quantified by thermogravimetry matches the expected aluminium fumarate chemical formula ([Al(OH)(Fum)]) and confirms full aluminium nitride conversion (Section S7 in the Supporting Information). IR spectroscopy reveals no significant difference between materials synthesized under solvothermal conditions and under vapour‐assisted conditions in the presence or absence of formic acid (Figure S3.9 in the Supporting Information). Besides the absence of residual crystalline AlN, PXRD indicates a change in the space group symmetry of the Al‐MIL‐53‐Fum product when the synthesis takes place in the presence of formic acid vapour (Figure 1, left). va‐Al‐MIL‐53‐Fum crystallises in the orthorhombic crystal system as indicated by a Pawley fit in the space group *Pnma* (Figure S4.5 in the Supporting Information). In contrast, in the absence of formic acid vapour (Figure S3.8 in the Supporting Information) or in solvothermal reactions (flow reactor and batch synthesis), a product crystallising in the monoclinic space group *P*2_1_/*c* is obtained, even when formic acid is used as a modulator in solution.^44^ va‐Al‐MIL‐53‐Fum has a BET surface area of 592 m^2^ g^−1^, which is much lower than the surface area of fr‐Al‐MIL‐53‐Fum (1000 m^2^ g^−1^) and that from the reported batch synthesis (1080 m^2^ g^−1^).^12^ However, the reduced porosity is ascribed to the different pore geometries of the different crystal structures. The pore size in va‐Al‐MIL‐53‐Fum (*Pnma*) is contracted in comparison to fr‐Al‐MIL‐53‐Fum (*P*2_1_/*c*). Positron Annihilation Lifetime Spectroscopy (PALS) measurements evidence this difference in the pore dimensions. For va‐Al‐MIL‐53‐Fum (*Pnma*), free‐volume elements with a diameter of 3.5 Å are observed, whereas for fr‐Al‐MIL‐53‐Fum (*P*2_1_/*c*) a much larger diameter of 5.9 Å is detected (Table S12.1 in the Supporting Information). These values are in good agreement with the size of the largest sphere that would fit in the pores, respectively, 4.1 Å and 5.8 Å, calculated by Monte Carlo sampling using Zeo++.^46^ Furthermore, simulations with RASPA indicate a difference in surface area of 32 % between va‐Al‐MIL‐53‐Fum (*Pnma*) and fr‐Al‐MIL‐53‐Fum (*P*2_1_/*c*), in line with the experimental data (41 %).^47^ As for Al‐MIL‐53‐Fum (*P*2_1_/*c*),^12^ no framework flexibility is observed for va‐Al‐MIL‐53‐Fum (Figure S3.6 in the Supporting Information). Still, alternative synthesis modulators to the corrosive formic acid are desirable for industrial production and should be further investigated.

#### Novel Al‐MIL‐53‐Mes material

**Figure 2:**
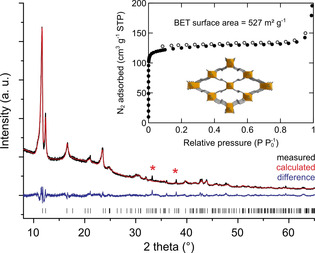
Al‐MIL‐53‐Mes prepared by vapour‐assisted synthesis: (main) Rietveld refinement from PXRD data; (inset) N_2_ physisorption isotherm and crystal structure. Red stars indicate traces of an unidentified crystalline impurity. Filled dots in the isotherm correspond to the adsorption branch, empty dots to the desorption branch.

By replacing fumaric acid with mesaconic acid, the optimized vapour‐assisted synthesis conditions with formic acid as modulator yield va‐Al‐MIL‐53‐Mes. The MIL‐53 type structure is formed under these conditions, in contrast to the MIL‐68 type product from flow and batch reactor syntheses (Figure 1, right). The crystal structure was confirmed by Rietveld refinement (Figure 2). Al‐MIL‐53‐Mes is a new microporous MOF material that crystallises in the orthorhombic space group *Pnma*, has a BET surface area of 527 m^2^ g^−1^ and a micropore volume of 0.169 cm^3^ g^−1^ (theoretical value: 0.210 cm^3^ g^−1^). The formation of Al‐MIL‐53‐Mes illustrates the potential of vapour‐assisted conditions to obtain new materials not accessible under solvothermal reaction conditions.

### Mixed‐linker MOFs with tuneable properties

**Figure 3:**
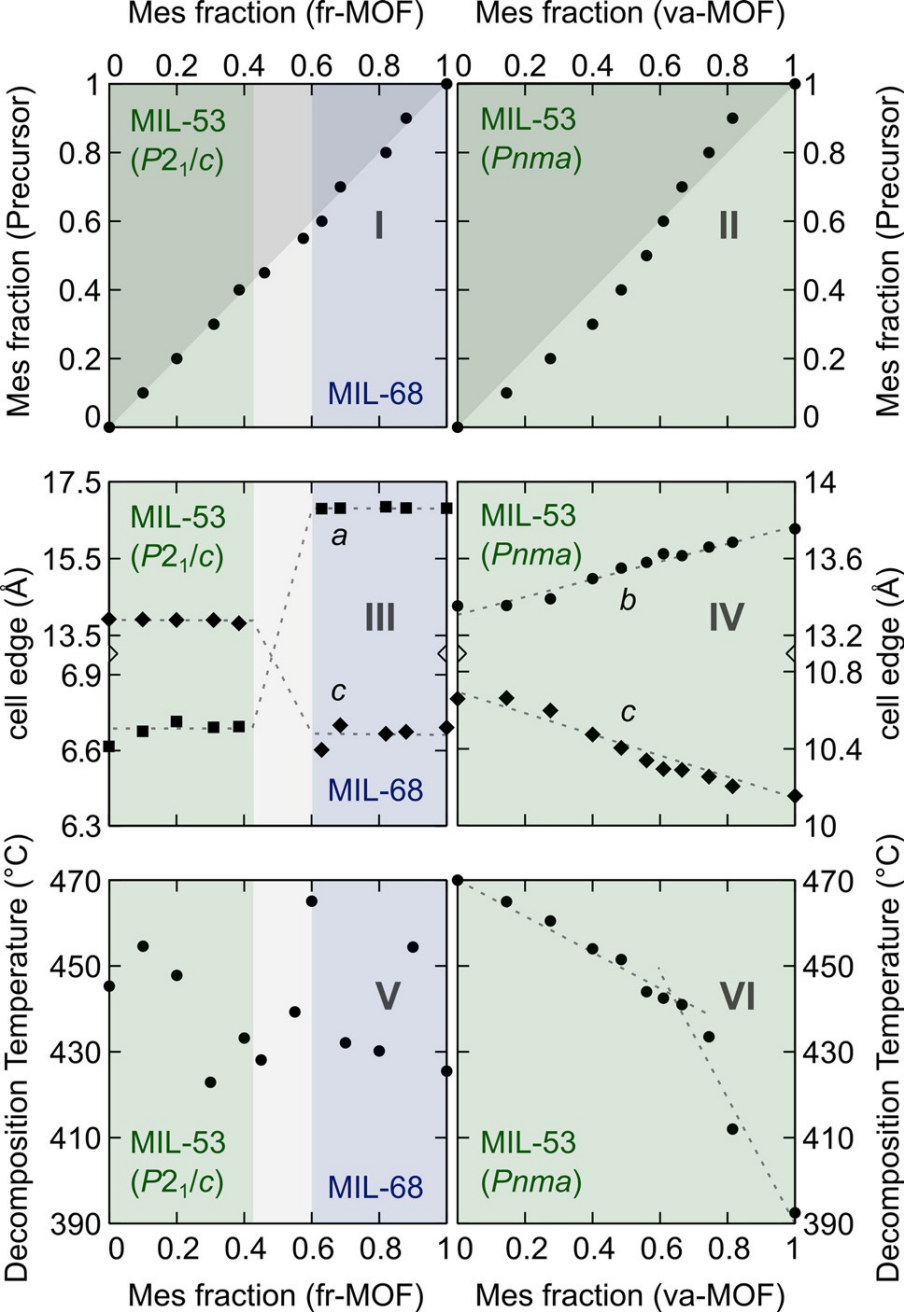
Mixed‐linker aluminium fumarate/mesaconate frameworks prepared by using the flow reactor (left) or vapour‐assisted synthesis (right) show properties dependent on the mesaconate content: (I, II) linker incorporation; (II, IV) unit cell dimensions and MOF topology; (V, VI) decomposition temperature. Coloured area and dashed lines are guides to the eyes. Only two unit cell edges are displayed for clarity, showing a discontinuity between two structure types (flow reactor) and a linear change characteristic of a solid solution in the material (vapour‐assisted).

The optimised fr‐ and va‐synthesis conditions for the single‐linker MOFs were used for the synthesis of mixed‐linker materials. For the fr‐syntheses, both linkers were dissolved in aqueous KOH. For the va‐synthesis, physical mixtures of the linker powders were used. The ratio of fumaric and mesaconic acid was varied from 0 to 100 % in 10 % steps for both approaches to obtain mixed‐linker MIL‐53 and MIL‐68 MOFs. IR spectroscopy shows the incorporation of both linkers in the framework (Section S8 in the Supporting Information). As for the single‐linker MOFs, elemental analysis shows only small impurities from the precursor, sulfur and nitrogen in the fr‐ and va‐products, respectively (Section S6 in the Supporting Information). ^1^H NMR spectroscopy confirms the absence of formate ions in the activated va‐products (Section S5 in the Supporting Information). All fr‐materials and va‐materials are large aggregates of crystallites smaller than 1 μm, with the crystallites from va‐synthesis having a more elongated shape and a larger size (Section S9 in the Supporting Information). The fraction of each linker was quantified by ^1^H NMR spectroscopy after dissolving the activated MOF. For the fr‐products, the linker ratio is close to the one in the precursor solution (<3 % deviation), meaning there is no preferential linker incorporation (Figure 3 I). The composition of the va‐products also follows the ratio in the linker powder mixture (<10 % deviation). However, a slight preference is observed for mesaconate or fumarate, respectively, below and above 65 % mesaconate content (Figure 3 II). At constant temperature and with excess solid linker present, the partial pressure of both H_2_Fum and H_2_Mes is independent of their ratio in the solid phase. Based on thermogravimetric measurements and the Knudsen effusion method, an approximately 4.5 times higher vapour pressure was determined for mesaconic acid at the reaction temperature (Section S11 in the Supporting Information). For an equilibrium reaction, products with a constant linker ratio are expected as long as each linker maintains its saturation vapour pressure (i.e., as long as solid linker is present). Nevertheless, as the incorporated linker ratio is not constant but varies with the solid linker mixture composition, it appears that the va‐synthesis of mixed‐linker MOFs is not an equilibrium reaction but rather under kinetic control. In other words, the rate of linker sublimation, which scales with the linker fraction in the reaction mixture, determines the composition of the mixed‐linker MOFs in the va‐route. Lastly, the organic content in the materials was quantified by thermogravimetry. For the va‐materials, the obtained values match very well the theoretical values, whereas for the fr‐materials they are generally lower, suggesting the slight presence of (hydr)oxide impurities (Tables S7.1 and S7.2 in the Supporting Information).

#### fr: the linker ratio directs the topology

**Figure 4:**
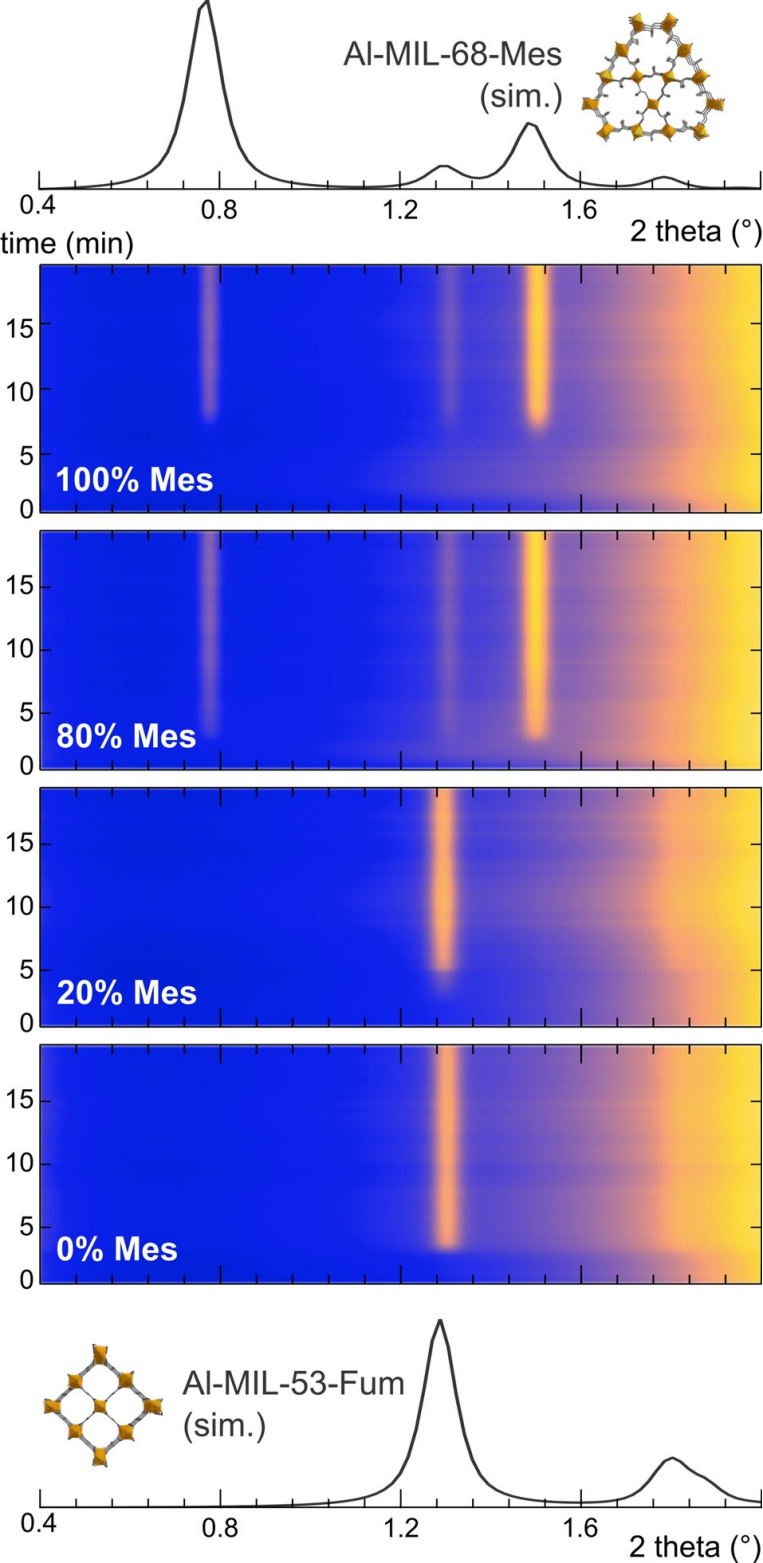
Results of the in situ PXRD experiment during hydrothermal batch synthesis of mixed‐linker materials. The final phase is formed directly from the synthesis solution, and is dictated by the linker ratio in the synthesis solutions, expressed as the mesaconate linker fraction. The simulated PXRD pattern for Al‐MIL‐53‐Fum (bottom) and Al‐MIL‐68‐Mes (top) are given for comparison. The X‐ray energy for the synchrotron experiments and simulations is 60 keV.

The fr‐products exhibit a MIL‐53 type structure at mesaconate contents below 40 %, and a MIL‐68 type structure above 60 % mesaconate. Thus, the linker present in the highest concentration determines the resulting framework topology in the fr‐route. Between 40 % and 60 % mesaconate, mixed phases are observed (Figure 3 III). In situ PXRD measurements in batch reactors show that for all linker ratios the final products crystallise directly from the precursor solution, without the formation of transient phases (Figure 4).

#### va: MIL‐53 topology at all linker ratios–solid solutions

Only the square‐grid MIL‐53 structure was observed for the va‐products, independent of the linker ratio. The lattice parameters *b* and *c* extracted from the position of the (0 1 1) reflection by Pawley fits change linearly with increasing mesaconate content (Figure 3 IV), whereas *a* remains constant (6.62±0.03 Å). According to Vegard′s law,^48^ the mixed‐linker MOFs obtained via va‐synthesis can thus be considered solid solutions. Conversely, the cell parameters of the fr‐products remained constant (Figure 3 III). However, as mentioned before, the va‐Al‐MIL‐53 materials crystallise in the orthorhombic space group *Pnma* whereas for the fr‐Al‐MIL‐53 materials the best fit is observed in the monoclinic space group *P*2_1_/*c*.

### Thermal stability

All materials show high thermal stability in air (>350 °C). For the fr‐materials, the decomposition temperature, calculated as the inflection point of the wt %–*T* curve upon decomposition, are in the range 425–465 °C (Figure 3 V), in line with the values observed by temperature‐dependent PXRD (Figure S2.4 in the Supporting Information). For the va‐Al‐MIL‐53 samples, a continuous decrease in decomposition temperature from approximately 470 to 395 °C is observed with increasing mesaconate content (Figure 3 VI). Between 65 % and 100 % mesaconate, the decomposition temperature drops more rapidly.

### Sorption properties and water cycling

**Figure 5:**
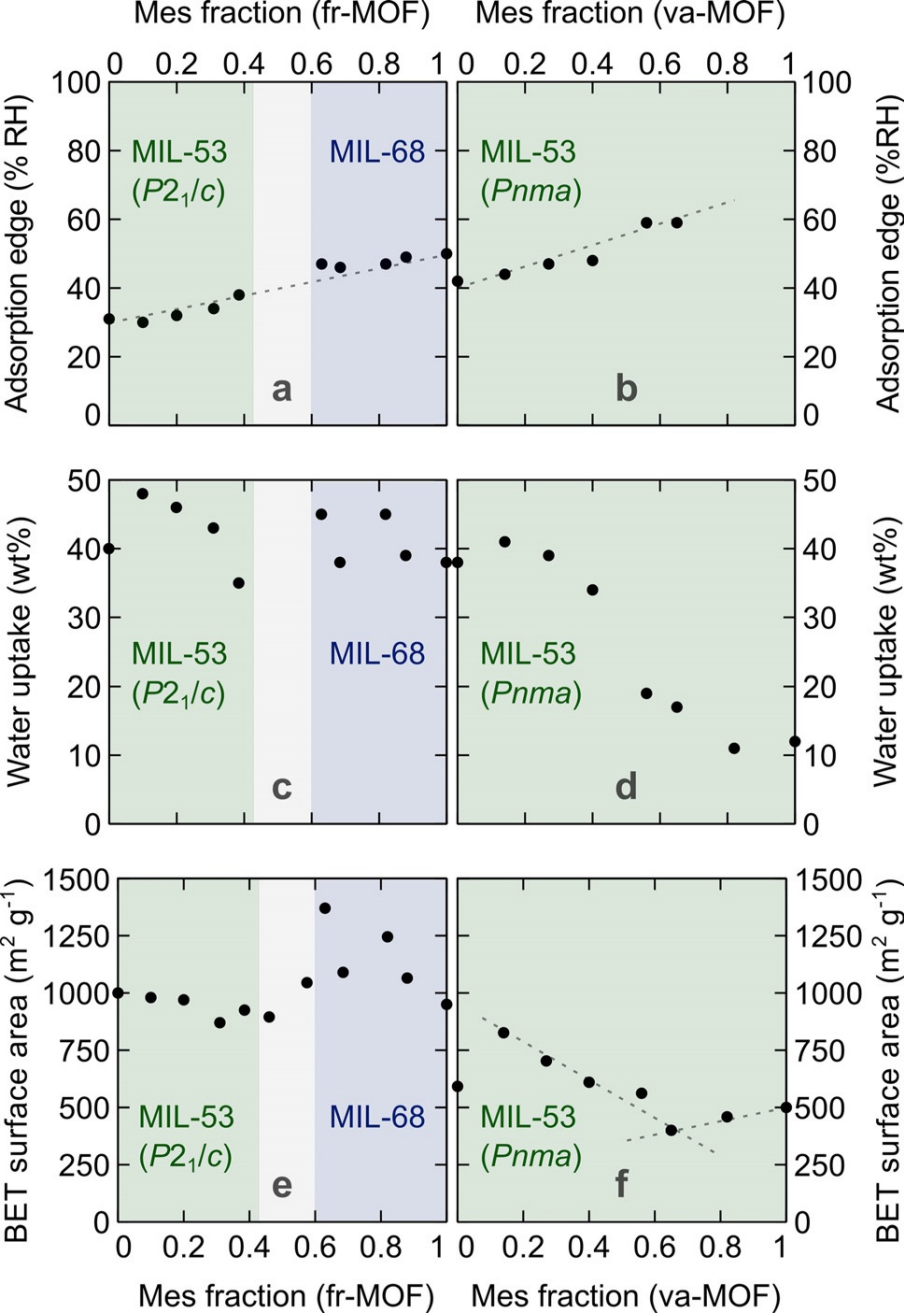
Mixed‐linker fumarate/mesaconate frameworks prepared by using the flow reactor (left) or vapour‐assisted synthesis (right) show tuneable water adsorption based on the mesaconate content: (a, b) adsorption edge of the volumetric water sorption isotherm; (c, d) gravimetric water uptake at 60 % relative humidity calculated from the volumetric water sorption isotherm; (e, f) BET surface area calculated from N_2_ sorption isotherms. The dashed lines are a guide to the eye.

**Figure 6:**
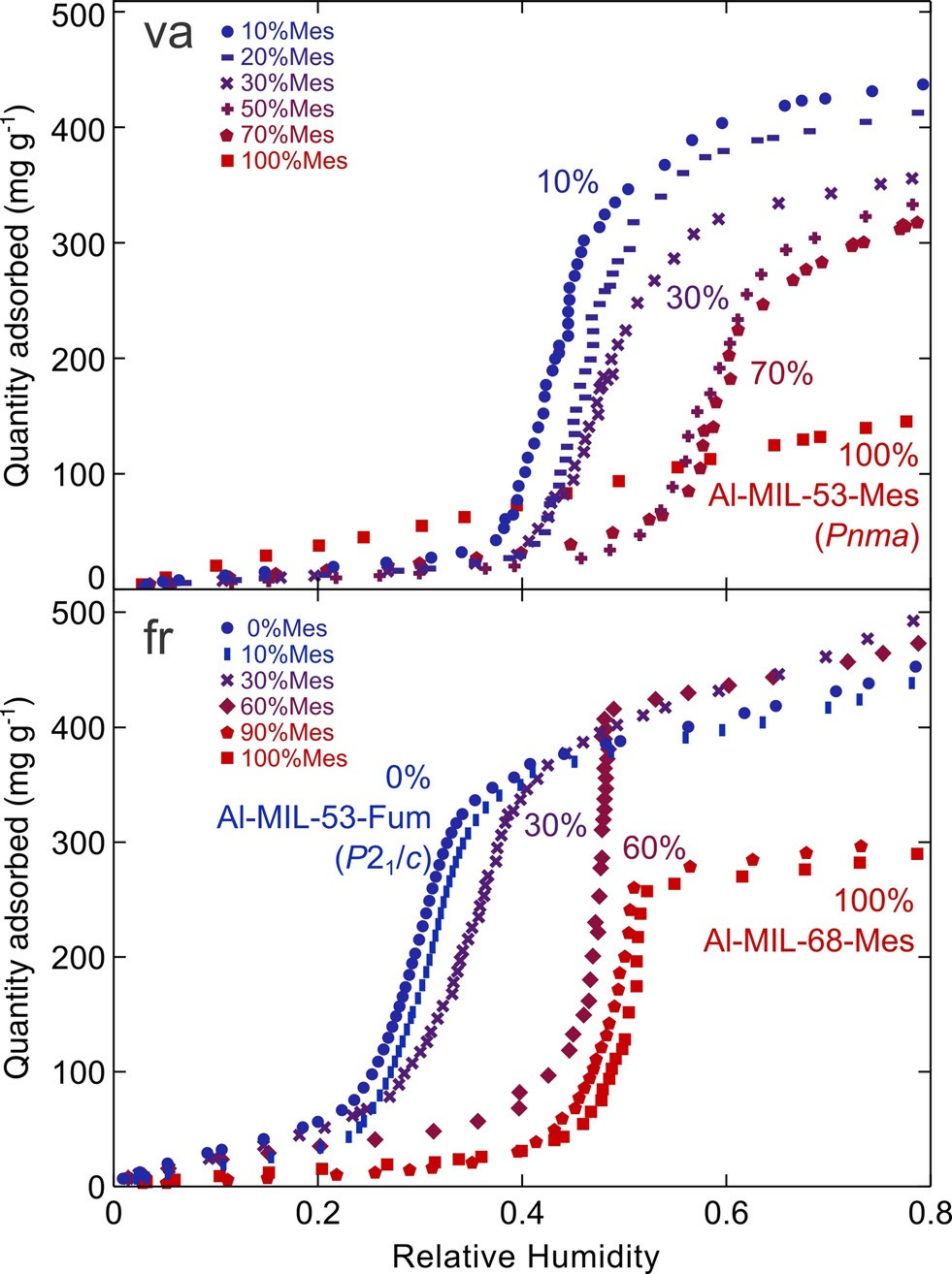
Water adsorption isotherms for mixed‐linker aluminium fumarate/mesaconate MOFs prepared by va‐synthesis (top) and fr‐synthesis (bottom). The volumetric isotherms were collected at 25 °C.

Water and nitrogen sorption measurements were carried out to investigate the effect of the crystal structure and the fraction of bulky and hydrophobic mesaconate linker on the sorption properties. The mixed‐linker fr‐materials with MIL‐53 type structure show a specific surface area comparable to Al‐MIL‐53‐Fum (approx. 1000 m^2^ g^−1^),^12^ yet slowly decrease with increasing mesaconate content (Figure 5). The surface area of the mixed‐linker fr‐Al‐MIL‐68 samples is slightly higher, as expected from literature data for Al‐MIL‐68‐Mes (1040 m^2^ g^−1^).^35^ Especially at 60 % and 80 % mesaconate, the materials exhibit a surprisingly but reproducibly high surface area of up to nearly 1400 m^2^ g^−1^ and a substantially higher decomposition temperature. With respect to the water uptake capacity, the fr‐materials show similar performance (42±5 wt % at 60 % RH), independent of the crystal structure (Figure 5 c). However, the influence of the increasing mesaconate content is clear from the shape of the water sorption isotherm. The sigmoidal isotherms show a sharp uptake at a specific relative pressure (Figure 6). This ‘adsorption edge’, defined as the RH value at the inflection point of the isotherm, shifts linearly from 30 to 50 % RH with increasing mesaconate content (Figure 5 a).

Similar to the single‐linker materials, mixed‐linker va‐Al‐MIL‐53 materials, crystallising in the orthorhombic space group *Pnma*, are porous but display lower surface areas than their monoclinic fr‐counterparts. Although a continuous decrease in surface area would be expected with increasing mesaconate content, a minimum is found at 65 % mesaconate (Figure 5 f). This composition corresponds to the most hydrophobic material as indicated by the highest adsorption edge at 59 % RH (Figure 5 b). At a mesaconate content lower than 65 %, the expected decrease of the adsorption edge and increase in water uptake capacity is observed (Figure 5 d). At a mesaconate content higher than 65 %, the water uptake capacity decreases and the isotherms gradually lose their sigmoidal shape, making it impossible to determine the adsorption edge (Figure 6).

**Figure 7:**
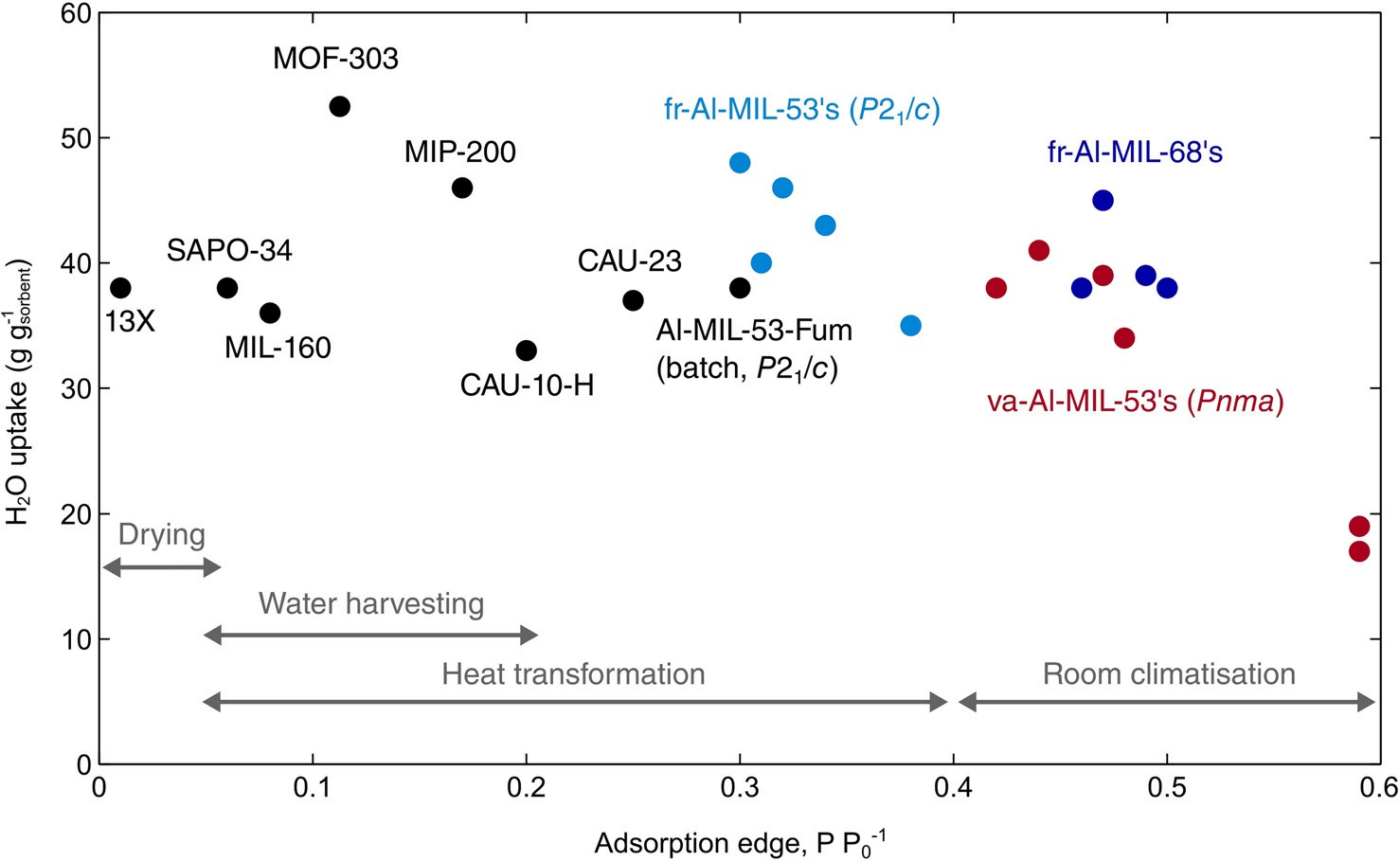
Comparison of fr‐ and va‐Al‐MOFs (experimental data, coloured markers) to best‐in‐class water adsorbents (literature values, black marker).^49^, ^50^ The water uptake at a relative pressure of 0.6 is plotted against the adsorption edge of the water adsorption isotherm measured at 25 °C. If the isotherm does not show a S‐shape, the relative pressure corresponding to half of the uptake is used instead. The adsorption edge desired range for several applications is also indicated.

The material sorption properties determine its application potential and which fields can be targeted. When compared with best‐in‐class water adsorbents, the va‐ and fr‐materials are competitive as they show comparable water uptakes. Further, the adsorption edge of the fr‐Al‐MIL‐53 materials (30–38 % RH), fr‐Al‐MIL‐68 materials (46–50 % RH) and va‐Al‐MIL‐53 materials (42–59 % RH) is comparatively higher (Figure 7),^49^, ^50^ and covers not only the desired range for heat transformation applications (5–40 % RH), but also the desired range for room climatisation (40–60 % RH).^51^

## Conclusion

Two potentially scalable synthesis methods were developed to obtain single and mixed‐linker aluminium dicarboxylate MOFs under mild conditions. Depending on the synthesis conditions, the crystallisation can be directed to different structure types, yielding materials with tuneable water sorption properties. These results will hopefully foster further research in the integration of MOFs in heat‐exchange or room climatisation devices. The discovery of a novel compound through vapour‐assisted synthesis indicates the opportunities in solvent‐free MOF synthesis and processing.

## Conflict of interest

The authors declare no conflict of interest.

## Supporting information

As a service to our authors and readers, this journal provides supporting information supplied by the authors. Such materials are peer reviewed and may be re‐organized for online delivery, but are not copy‐edited or typeset. Technical support issues arising from supporting information (other than missing files) should be addressed to the authors.

Supplementary
