# Supplementary material for: Aqueous Flow Reactor and Vapour‐Assisted Synthesis of Aluminium Dicarboxylate Metal–Organic Frameworks with Tuneable Water Sorption Properties
Source: Chemistry. 2020 Jul 27;26(47):10841–8. doi: 10.1002/chem.202001661 (PMC7496088; doi:10.1002/chem.202001661)
Supplement: Supplementary file 1 — Supplementary [file CHEM-26-10841-s001.pdf]

Chemistry–A European Journal

Supporting Information

Aqueous Flow Reactor and Vapour-Assisted Synthesis of Aluminium Dicarboxylate Metal–Organic Frameworks with Tuneable Water Sorption Properties**

Timothée Stassin+,[a] Steve Waitschat+,[b] Niclas Heidenreich,[b] Helge Reinsch,[b] Finn Pluschkell,[b] Dmitry Kravchenko,[a] João Marreiros,[a] Ivo Stassen,[a] Jonas van Dinter,[b] Rhea Verbeke,[a] Marcel Dickmann,[c] Werner Egger,[d] Ivo Vankelecom,[a] Dirk De Vos,[a] Rob Ameloot,*[a] and Norbert Stock*[b]

Aqueous flow reactor and vapour-assisted synthesis of aluminium dicarboxylate metal-organic frameworks with tuneable water sorption properties

Timothée Stassin,#[a] Steve Waitschat,#[b] Niclas Heidenreich,[b] Helge Reinsch,[b] Finn Pluschkell,[b] Dmitry Kravchenko,[a] João Marreiros,[a] Ivo Stassen,[a] Jonas van Dinter,[b] Rhea Verbeke,[a] Marcel Dickmann,[c] Werner Egger,[d] Ivo Vankelecom,[a] Dirk De Vos,[a] Rob Ameloot*[a] and Norbert Stock*[b]

Contents

S1. Methods & Reagents 2

S2. Flow reactor (fr) Synthesis of fr-Al-MIL-53-Fum, fr-Al-MIL-68-Mes and Mixed-Linker fr-Al-MIL-53/68-Fum/Mes 4

S2.1. Flow Reactor Synthesis of Al-MIL-53-Fum 4

S2.2. Flow Reactor Synthesis of Al-MIL-68-Mes 5

S2.3. Flow Reactor Synthesis of Mixed-Linker Al-MIL-53/68-Fum/Mes 6

S3. Vapour-Assisted (va) Synthesis of Al-MIL-53-Fum, Al-MIL-53-Mes and Mixed-Linker Al-MIL-53-Fum/Mes 8

S3.1. Vapour-Assisted Synthesis of Al-MIL-53-Fum 8

S3.2. Vapour-Assisted Synthesis of Al-MIL-53-Mes and Mixed-Linker Al-MIL-53-Fum/Mes 15

S4. Structure Refinement, Le Bail and Pawley Fits 16

S5. ^{1}H NMR Spectroscopy 21

S6. Elemental Analyses 23

S7. Thermogravimetric Analyses 24

S8. Fourier-Transform Infrared Spectroscopy 26

S9. Scanning Electron Microscopy 28

S10. Nitrogen Physisorption 35

S11. Vapour pressure determination of mesaconic acid and fumaric acid via thermogravimetry 38

S12. Positronium Annihilation Lifetime Spectroscopy 41

S13. References 42

S1. Methods & Reagents

Powder X-ray Diffraction (PXRD)

Initial characterisation was performed using a Stoe Stadi P diffractometer fitted with an xy-stage, in transmission geometry using Cu $K\alpha_1$ radiation and with data collected by an image plate detector, or a STOE COMBI P diffractometer (monochromated Cu $K\alpha_1$) equipped with an IP-PSD detector in transmission geometry.

Powder X-Ray diffraction (PXRD) patterns for Pawley fitting and Rietveld refinement were measured using a Stoe Stadi P diffractometer in transmission geometry using Cu $K\alpha_1$ radiation and with data collected using a Mythen detector.

For temperature-dependent PXRD, the samples were prepared in 0.5 mm silica capillaries, which were then heated up. PXRD patterns were measured every 10 °C for five minutes on a Stoe Stadi P diffractometer in transmission geometry using Mo $K\alpha_1$ radiation.

Fourier Transform Infrared Spectroscopy (FTIR)

Infrared spectra were recorded (64 scans, 4 cm^{-1} resolution) over the spectral range 400-4000 cm^{-1} on a Bruker ALPHA-P A220/D-01 FTIR spectrometer fitted with an ATR unit or on a Varian 670 FTIR spectrometer attached to a Varian 620 FTIR microscope equipped with a slide-on Ge ATR tip.

Thermogravimetric Analysis (TGA)

Thermogravimetric analysis was carried out in air using a Netzsch STA 449 F3 Jupiter thermogravimetric analyser (5 °C min^{-1} heating rate).

Elemental Analysis

Elemental analysis was performed using a EuroVector EuroEA elemental analyser.

Nuclear Magnetic Resonance (NMR) Spectroscopy

^{1}H NMR spectroscopy was performed using a Bruker DRX 500 spectrometer or a Bruker Avance 600 spectrometer.

Scanning Electron Microscopy (SEM)

SEM images were collected using a Philips XL30 FEG. The samples were sputter coated with 5 nm of Pt before the analysis.

Nitrogen and Water Physisorption

N_2 sorption isotherms were recorded at -196 °C with a BELSORP-mini apparatus (BEL Japan Inc.) or with a Micrometrics 3Flex gas physisorption instrument. Water sorption isotherms were recorded at 25 °C with a BELSORP-max apparatus (BEL Japan Inc.). The samples were degassed at 130-150 °C in vacuum prior to measurement.

Water uptake cycling

Cycled gravimetric water uptake measurements were performed using a water-filled bubbler attached to a Netzsch STA 449 F3 Jupiter thermogravimetric analyser. Under continuous humid Ar flow, about 15 mg of material was inserted in the instrument furnace and the temperature was cycled according to the following sequence: 5 °C min^{-1} heating rate – isothermal step (at 120 or 150 °C) – convective cooling – isothermal step (at 30 °C).

In situ crystallisation

In situ crystallisation experiments were performed using a multi-purpose reaction cell for the investigation of reactions under solvothermal conditions.[1] The experiments were carried out at the light source PETRA III at DESY. The syntheses were performed under batch conditions instead of continuous flow. For the *in situ* batch synthesis of Al-MIL-53/68-Fum/Mes, 1.5 ml of a 0.05 M aluminium sulfate aqueous solution was mixed with 1.5 ml of a 0.1 M linker 0.3 M NaOH aqueous solution in the reaction tube and heated.

Positron Annihilation Lifetime Spectroscopy (PALS)

Positron Annihilation Lifetime Spectroscopy (PALS) with monochromatic pulsed beams of variable energy allows to measure the free-volume elements in porous materials. It is based on the annihilation of the ortho-positronium (o-Ps) with an electron of its surroundings. The free-volume element size can be extracted from the average lifetime of the o-Ps via the Tao-Eldrup model[2,3], which assumes spherical holes with infinite walls:

$$\tau_3 = \frac{1}{2}[1 - \frac{R}{R+\Delta R} + \frac{1}{2\pi}\sin(\frac{2\pi R}{R+\Delta R})]^{-1} \text{ [ns]}$$

with τ_3 the o-Ps lifetime, R the radius of the free-volume element and ΔR the thickness of the electron layer surrounding the free-volume element, which is assumed to be 0.166 nm.

The pulsed low energy positron system (PLEPS) at the neutron induced positron source Munich (NEPOMUC) was used for these measurements.[4] All powder samples were measured at a positron implantation energy of 1.5 keV, corresponding to a mean implantation depth of 56.45, 49.87 and 43.05 nm, assuming a density of 1.06 g cm^{-3} (fr-Al-MIL-53-Fum, space group $P2_1/c$) and 1.20 g cm^{-3} (va-Al-MIL-53-Fum, space group *Pnma*), respectively. A time resolution of 250 ps was achieved and at least 4 million counts were collected for each spectra, with a count rate of 10 000 cts s^{-1}. The resolution function was determined via p-doped SiC, with known positron lifetimes. The spectra were deconvoluted into four or five lifetimes (i.e. p-Ps, free e$^+$, two or three o-Ps) with the software PALSfit3.[5] The reported diameters are based on the shortest o-Ps lifetimes.

Reagents

Table S1.1 List of reagents and suppliers used in the flow reactor (fr) synthesis of fr-Al-MIL-53-Fum, fr-Al-MIL-68-Mes and fr-Al-MIL-53/68-Fum/Mes.

Reagent	Supplier	Reagent	Supplier
$Al_2(SO_4)_3{\cdot}18H_2O$	Grüssing	Fumaric acid	Sigma-Aldrich
Sodium hydroxide	Grüssing	Mesaconic acid	Sigma-Aldrich
Ethanol	Walter CMP		

Table S1.2 List of reagents and suppliers used in the vapour-assisted synthesis of va-Al-MIL-53-Fum, va-Al-MIL-53-Mes and va-Al-MIL-53-Fum/Mes.

Reagent	Supplier	Reagent	Supplier
Aluminium nitride	Abcr	Fumaric acid	Janssen chimica
Formic acid	Sigma-Aldrich	Mesaconic acid	Sigma-Aldrich

S2. Flow reactor (fr) Synthesis of fr-Al-MIL-53-Fum, fr-Al-MIL-68-Mes and Mixed-Linker fr-Al-MIL-53/68-Fum/Mes

S2.1. Flow Reactor Synthesis of Al-MIL-53-Fum

For the syntheses of fr-Al-MIL-53-Fum, a 0.05 M aqueous solution of aluminium sulfate ($Al_2(SO_4)_3 \cdot 18H_2O$) and a 0.1 M aqueous solution of the linker fumaric acid (H_2Fum) mixed with NaOH (1:3) were prepared. These solutions were loaded into the syringe pumps and connected to the flow reactor in a three-pump configuration, where the third syringe filled with water was used to push the product out of the reactor. The used reactor is similar to the reactor published by Waitschat et al.[6] with a reactor volume of approximately 14.28 ml. For synthesis optimisation different reaction times (10, 15 and 30 min) and metal-to-linker ratios (2:1, 1:1, 1:2) were tested, as regulated by the flow rate of the single syringes. The product was collected in an ice-cooled beaker to rapidly cool down the reaction mixture. Afterwards the suspension was centrifuged, the solid fraction washed with EtOH and eventually dried at 70 °C.

Table S2.1 Reaction parameters of the synthesis optimisation of Al-MIL-53-Fum using a three-pump flow reactor, with water as the transport medium. The optimised reaction parameters are marked by a red box; a = pump flow rate.

Al^{3+} a / mL min^{-1}	Fum^{2-} a / mL min^{-1}	T / °C	t / min	Product
0.952	0.476	80	10	No product
0.714	0.714	80	10	Al-MIL-53-Fum
0.476	0.952	80	10	Low crystallinity
0.635	0.318	80	15	No product
0.476	0.476	80	15	Al-MIL-53-Fum
0.318	0.635	80	15	Low crystallinity
0.317	0.159	80	30	Low crystallinity
0.238	0.238	80	30	Al-MIL-53-Fum
0.159	0.317	80	30	Low crystallinity

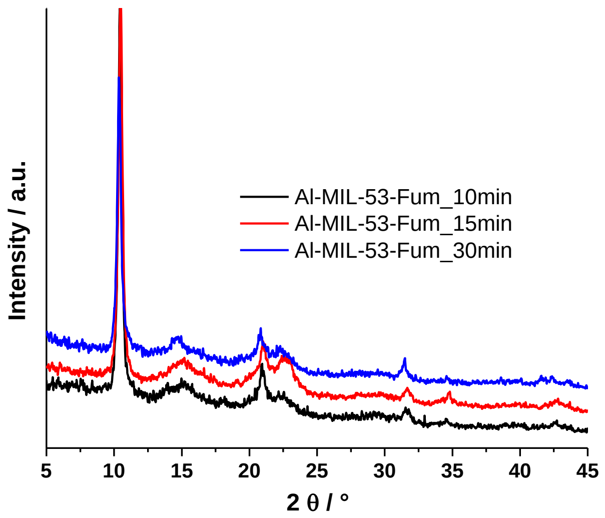

Figure S2.1 PXRD patterns of fr-Al-MIL-53-Fum prepared in the flow reactor with the optimised metal-to-linker ratio of 1:1 and various reaction times.

S2.2. Flow Reactor Synthesis of Al-MIL-68-Mes

For the syntheses of fr-Al-MIL-68-Mes, a 0.05 M aqueous solution of aluminium sulfate ($Al_2(SO_4)_3 \cdot 18H_2O$) and a 0.1 M aqueous solution of the linker mesaconic acid (H_2Mes) mixed with NaOH (1:3) were prepared. These solutions were loaded into the syringe pumps and connected to the flow reactor in a three-pump configuration, where the third syringe filled with water was used to push the product out of the reactor. The used reactor is similar to the reactor published by Waitschat et al.[6] with a reactor volume of approximately 14.28 ml. For synthesis optimisation the optimised metal to linker ratio of 1:1 was fixed and only the reaction time was varied (10, 15 and 30 min). The product was collected in an ice-cooled beaker to rapidly cool down the reaction mixture. Afterwards the suspension was centrifuged, the solid fraction washed with EtOH and eventually dried at 70 °C.

Table S2.2 Reaction parameters of the synthesis optimisation of fr-Al-MIL-53-Fum using a three-pump flow reactor, with water as the transport medium. The optimised reaction parameters are marked by a red box; a = pump flow rate.

Al^{3+} a / ml min^{-1}	Mes^{2-} a / ml min^{-1}	T / °C	t / min	Product
0.714	0.714	80	10	No product
0.476	0.476	80	15	Al-MIL-68-Mes
0.238	0.238	80	30	Al-MIL-68-Mes

S2.3. Flow Reactor Synthesis of Mixed-Linker Al-MIL-53/68-Fum/Mes

For the syntheses of mixed-linker fr-Al-MIL-53-Fum/Mes and fr-Al-MIL-68-Fum/Mes, the optimised reaction conditions of Al-MIL-53-Fum and Al-MIL-68-Mes were combined. Thus, a 0.05 M aqueous solution of aluminium sulfate ($Al_2(SO_4)_3 \cdot 18H_2O$) was prepared. An aqueous solution of the linkers with a 0.1 M total concentration, and NaOH (1:3), was also prepared. Only the fraction of fumaric acid and mesaconic acid was varied, from 0% to 100% in 10% steps. All other parameter (e.g. pump flow rates) were kept constant and identical to the optimal values for the single linker compounds.

Table S2.3 Reaction parameters of the synthesis of mixed linker fr-Al-MIL-53/68-Fum/Mes using a three-pump flow reactor, with water as the transport medium; a = pump flow rate.

H_2Fum / g	H_2Mes / g	H_2Fum : H_2Mes	Al^{3+} a / ml min^{-1}	T / °C	t / min	Product
0.0116	0.1170	10 : 90	0.476	80	15	Al-MIL-68_90%Mes
0.0232	0.1040	20 : 80	0.476	80	15	Al-MIL-68_80%Mes
0.0348	0.0910	30 : 70	0.476	80	15	Al-MIL-68_70%Mes
0.0464	0.0780	40 : 60	0.476	80	15	Al-MIL-68_60%Mes
0.0522	0.0715	45 : 55	0.476	80	15	Al-MIL-68_55%Mes
0.0638	0.0585	55 : 45	0.476	80	15	Al-MIL-53_45%Mes
0.0696	0.0520	60 : 40	0.476	80	15	Al-MIL-53_40%Mes
0.0812	0.0390	70 : 30	0.476	80	15	Al-MIL-53_30%Mes
0.0928	0.0260	80 : 20	0.476	80	15	Al-MIL-53_20%Mes
0.1044	0.0130	90 : 10	0.476	80	15	Al-MIL-53_10%Mes

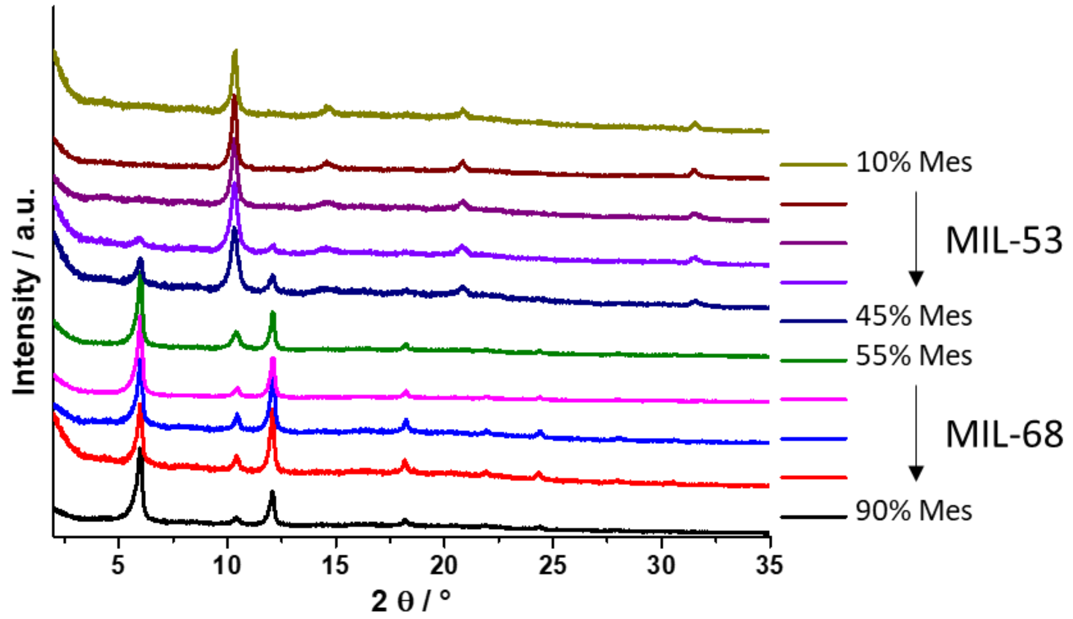

Figure S2.2 PXRD patterns of the mixed-linker fr-Al-MIL-53/68-Fum/Mes prepared in the flow reactor. The crystal structure changes between 45% and 55% mesaconate content from the MIL-53 type to the MIL-68 type structure.

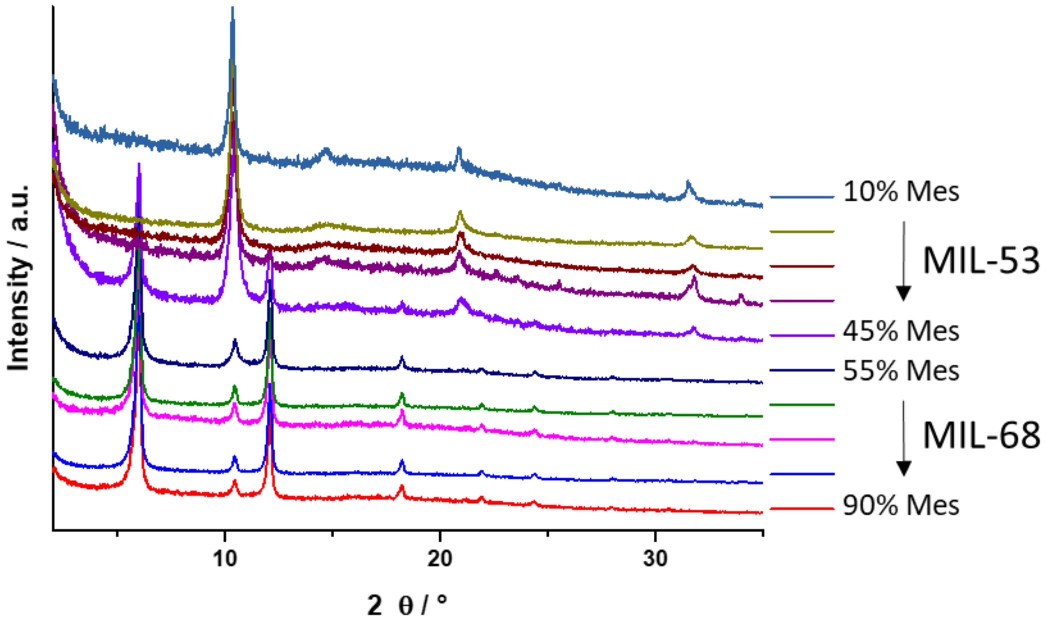

Figure S2.3 PXRD patterns of the replicates of mixed-linker fr-Al-MIL-53/68-Fum/Mes prepared in the flow reactor, showing the same structural changes between 45% and 55% mesaconate content as shown in Figure S2.3.

Temperature-dependent PXRD

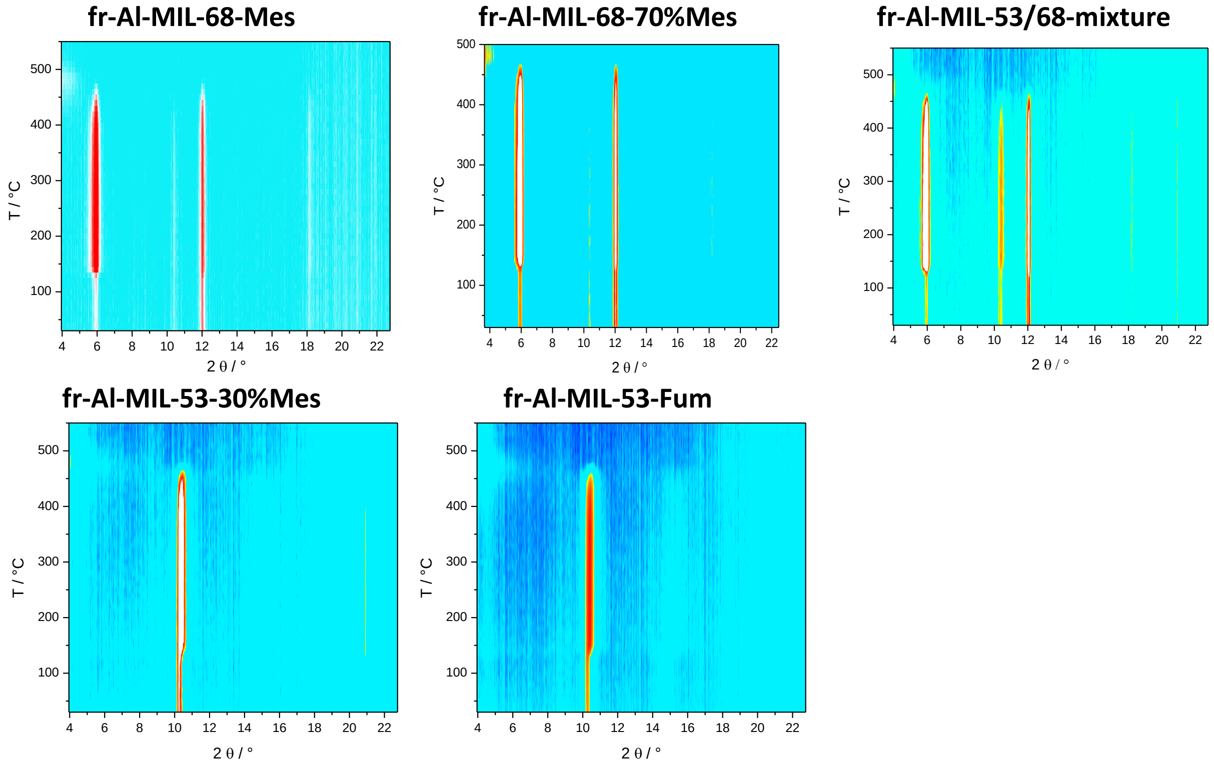

Figure S2.4 Results of temperature-dependent PXRD measurements of fr-Al-MIL-68 and fr-Al-MIL-53 materials with various mesaconate content. All samples show a similar decomposition temperature of approximately 460 °C.

S3. Vapour-Assisted (va) Synthesis of Al-MIL-53-Fum, Al-MIL-53-Mes and Mixed-Linker Al-MIL-53-Fum/Mes

S3.1. Vapour-Assisted Synthesis of Al-MIL-53-Fum

Reaction conditions overview

For the vapour-assisted (va) syntheses of va-Al-MIL-53-Fum, powder physical mixtures of the aluminium source and the fumaric acid were prepared. About 300 mg powder physical mixture was placed along smaller glass vial(s) containing the liquid additive(s) in a 25 mL glass bottle with PTFE-protected caps. The sealed bottle was placed in an oven preheated at 80 °C and left to react isothermally for 48 h. Afterwards, the bottle was removed from the oven, immediately opened and the liquid-containing vial(s) removed. The powder sample in the bottle dried for 1 h in the oven at 80 °C. Subsequently, the excess ligand was removed through sublimation by heating the sample in vacuum (heating rate 5 °C min^{-1}) at 200 °C for 2 h. Eventually, the material was annealed (heating rate 3 °C/min) at 300 °C for 12 h.

Table S3.1 Reaction details of the vapour-assisted synthesis optimisation of va-Al-MIL-53-Fum. The optimised reaction conditions are marked by a red box.

#	Al^{3+} source	Al^{3+}: H_2Fum / molar	Additive(s)[a]	Product
1	Al_2O_3	1:2	-	No product
2	AlN	1:2	-	No product
3	AlN	1:2	Water	va-Al-MIL-53-Fum + AlN
4	AlN	1:2	Sat. K_2SO_4	va-Al-MIL-53-Fum + AlN
5	AlN	1:2	Sat. KCl	No product
6	AlN	1:2	Sat. $(NH_4)_2$SO4	No product
7	AlN	1:2	Sat. NaCl	No product
8	AlN	1:2	FA, Sat. K_2SO_4	va-Al-MIL-53-Fum
9	AlN	1:2	FA, Sat. KCl	va-Al-MIL-53-Fum

[a]deionised water, formic acid (FA), or saturated salt solutions in water.

Attempts to the solvent-free synthesis of Al-MIL-53-Fum

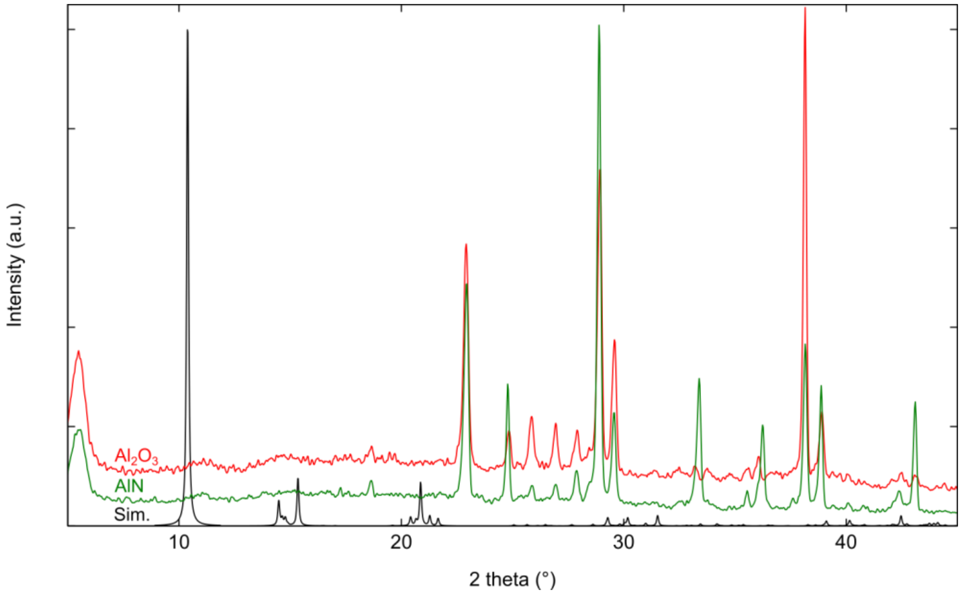

Figure S3.1 PXRD patterns of reaction products obtained using AlN or Al_2O_3 in solvent free reactions with H_2Fum. The Al-MIL-53-Fum phase (simulated pattern, black) is not formed upon heating of a physical mixture of alumina (red) or aluminium nitride (green) and fumaric acid at 80 °C for 48 h, i.e. entries 1-2 in Table S3.1. X-ray diffractograms of the samples as synthesized.

Attempts to the vapour-assisted synthesis of Al-MIL-53-Fum under various relative humidities

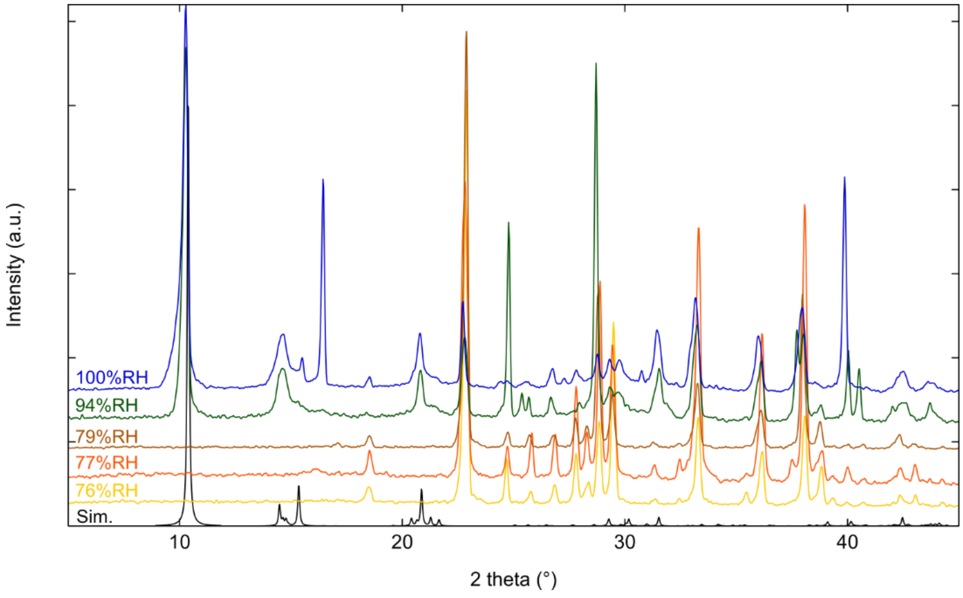

Figure S3.2 PXRD patterns of different reaction products obtained in va-reactions. The Al-MIL-53-Fum phase (simulated pattern, black) only forms at high relative humidity values: X-ray diffractogram of the samples after heating physical mixtures of AlN and H_2Fum in presence of water (100% RH, blue), saturated K_2SO_4 solution (94% RH, green), saturated KCl solution (79% RH, brown), saturated $(NH4)_2SO_4$ solution (77% RH, orange), or saturated NaCl solution (76% RH, yellow), i.e. entries 3-7 in Table S3.1, at 80 °C for 48 h.

Thermal activation of Al-MIL-53-Fum prepared via vapour-assisted synthesis

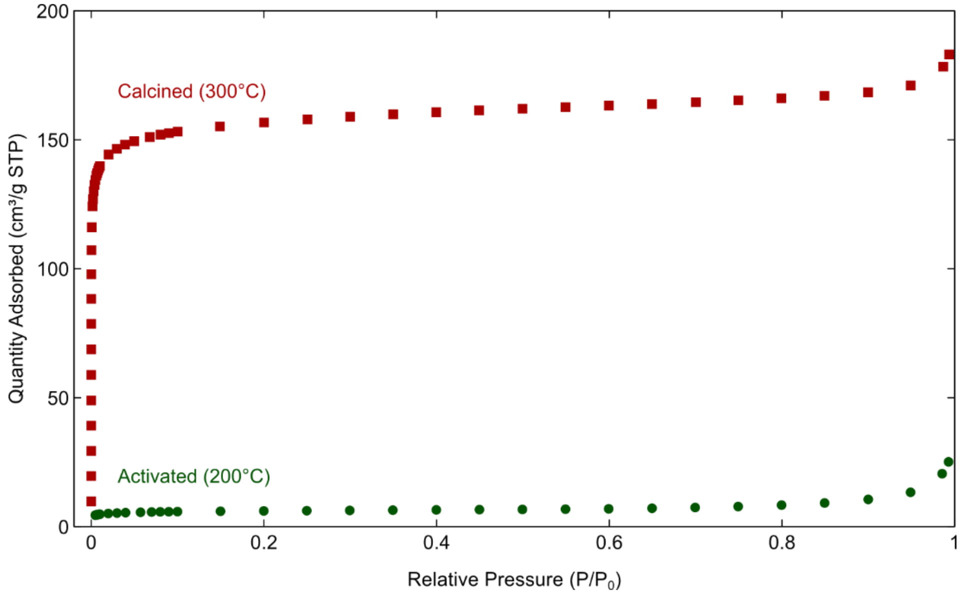

Figure S3.3 N_2 sorption isotherms of va-Al-MIL-53-Fum (entry 3 Table S2.3), collected at 77 K. Calcination at 300 °C for 12 h (red) is necessary to retrieve the microporosity of the material, while heating the as synthesized material in vacuum at 200 °C for 2 h (green) is not sufficient. Analysis of the N_2 adsorption isotherm of the annealed material (i.e. mixture of va-Al-MIL-53-Fum and unreacted AlN) following the Rouquerol criteria[7] yields a micropore surface area of 548 $m^2\ g^{-1}$ (BET range 0.004-0.04 P P_0^{-1}) and an external surface area of 78 $m^2\ g^{-1}$ (t-plot, Harkins and Jura, thickness range 3.5-5 Å).

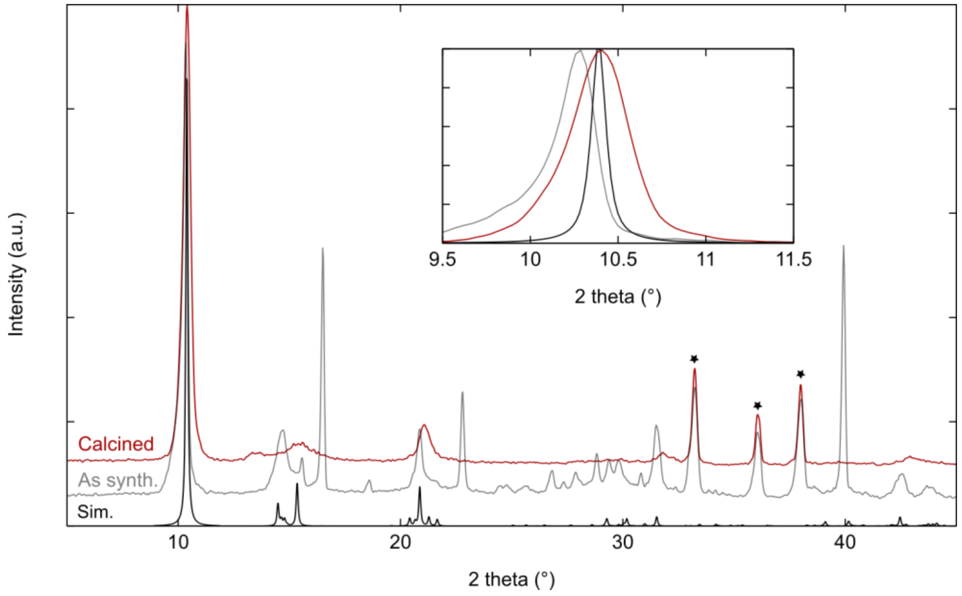

Figure S3.4 PXRD patterns of va-Al-MIL-53-Fum (entry 3 in Table S3.1) before and after solvent-free thermal activation. By heating in vacuum at 200 °C for 2 h and subsequent calcination at 300 °C for 12 h (red), the excess and trapped ligand present in the as synthesized material (grey) can be removed, resulting in a mixture of as-Al-MIL-53-Fum (simulated, black) and unreacted aluminium nitride (black stars). The insert shows a magnification of the 9.5-11.5 °2 theta range where a shift of the Al-MIL-53 001 reflection can be observed upon calcination indicating small changes in the framework as often and to a larger extent observed for many MIL-53 type compounds.

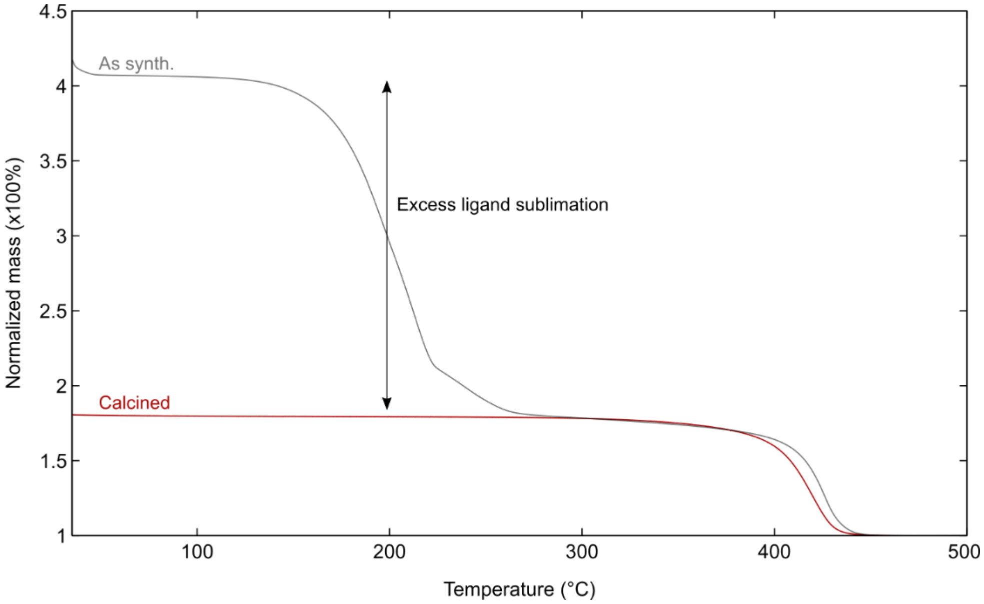

Figure S3.5 TG curve of va-Al-MIL-53-Fum (entry 3 in Table S3.1) before and after solvent-free thermal activation. Heating in vacuum at 200 °C is effective at subliming the excess ligand present in the as synthesized material (grey). The similar TGA traces at higher temperatures of the as synthesized material (grey) and material after calcination at 300 °C for 12 h (red), further indicate the amount of trapped ligand to be minimal.

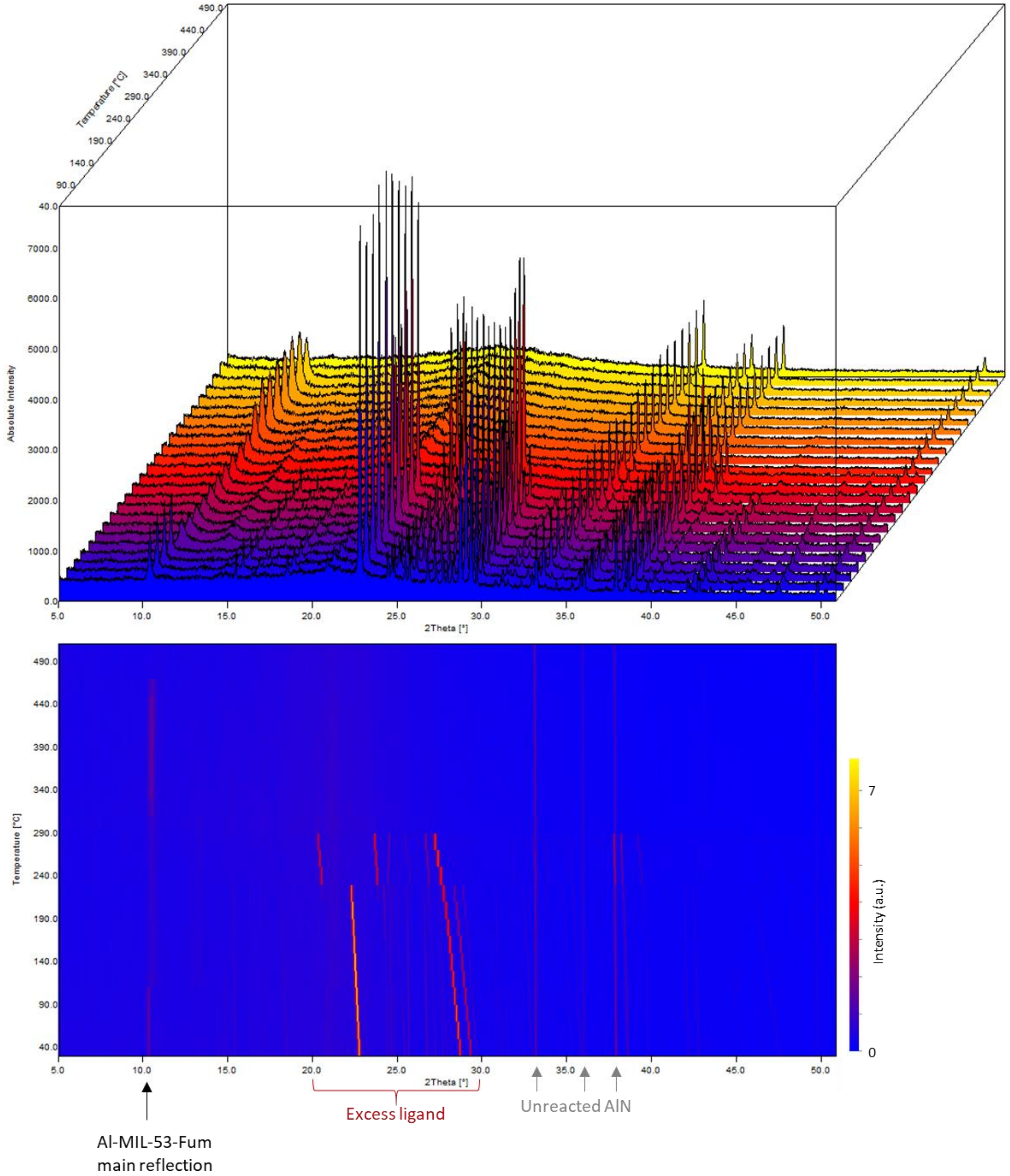

Figure S3.6 Temperature-dependent X-ray diffractograms of the as synthesized va-Al-MIL-53-Fum material reveals a disappearance of the ligand reflections between 40 and 300 °C , thereby confirming heat treatment to be effective for removal of excess ligand by sublimation.

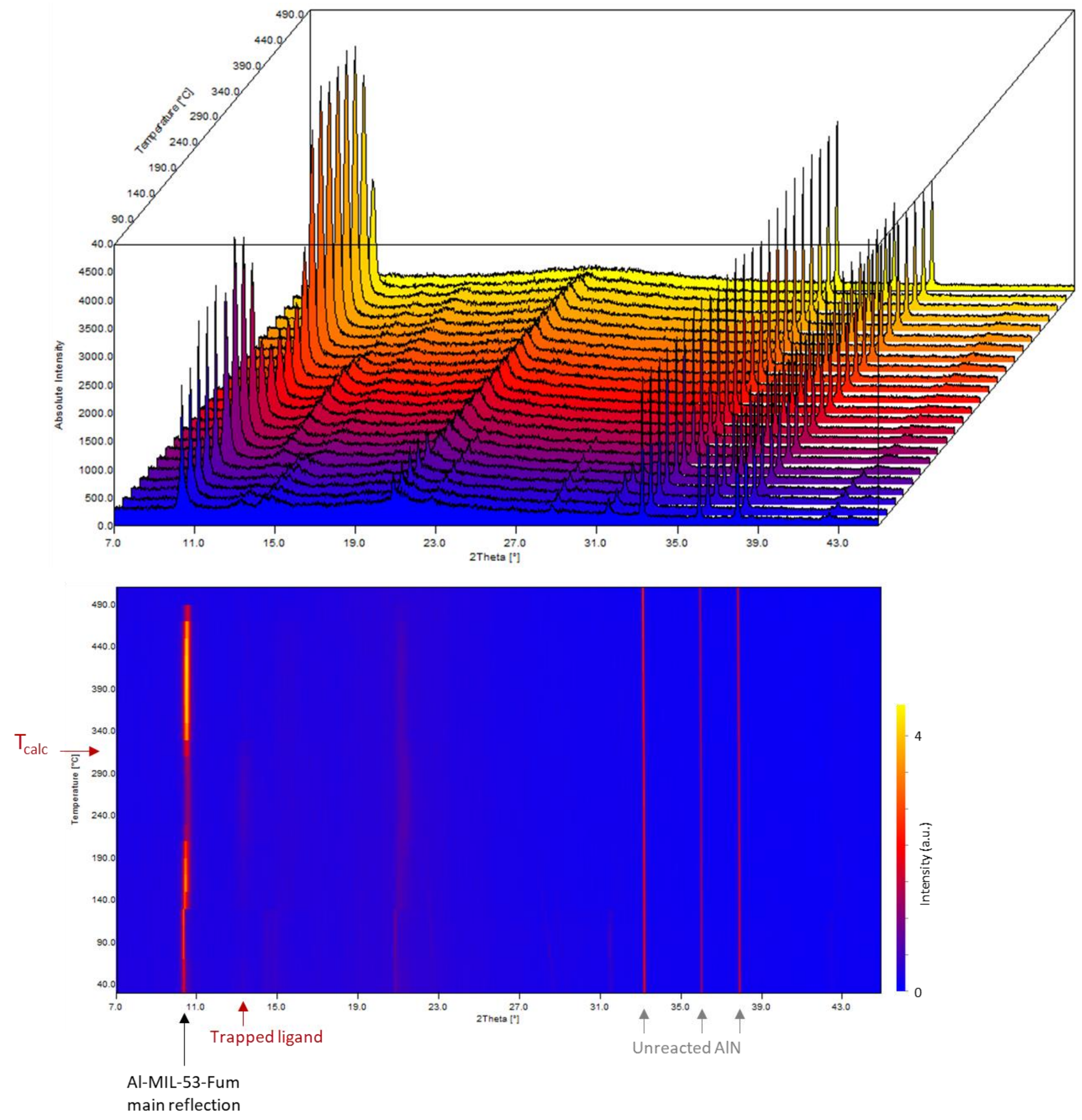

Figure S3.7 Temperature-dependent X-ray diffractograms of the va-Al-MIL-53-Fum material with prior ligand sublimation (right) evidence the effect of calcination: disappearance around 300 °C of a reflection assigned to linker molecules trapped in the pores, leaving only Al-MIL-53-Fum and aluminium nitride reflections at higher temperatures.

Formic acid vapour ensures full aluminium nitride hydrolysis

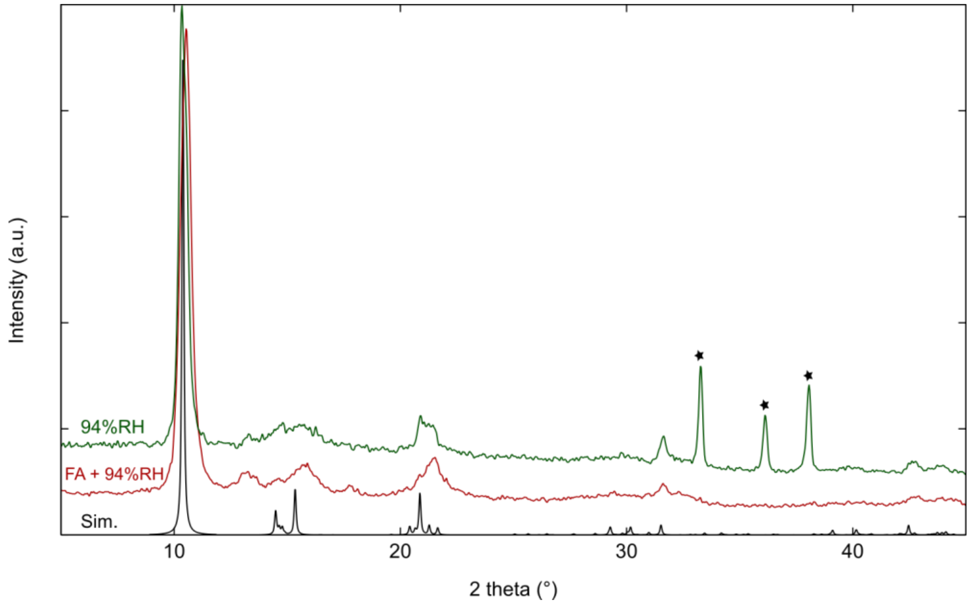

Figure S3.8 PXRD patterns of Al-MIL-53-Fum materials prepared via vapour-assisted synthesis in presence (red) and absence (green) of formic acid (FA) vapour (entries 4 and 8 in Table S3.1). The presence of formic acid vapour in the reactor results in (i) full hydrolysis of the aluminium nitride (black stars) and change in the Al-MIL-53-Fum structure from space group $P2_1/c$ (simulated, black) to *Pnma* (see also Figure S4.5).

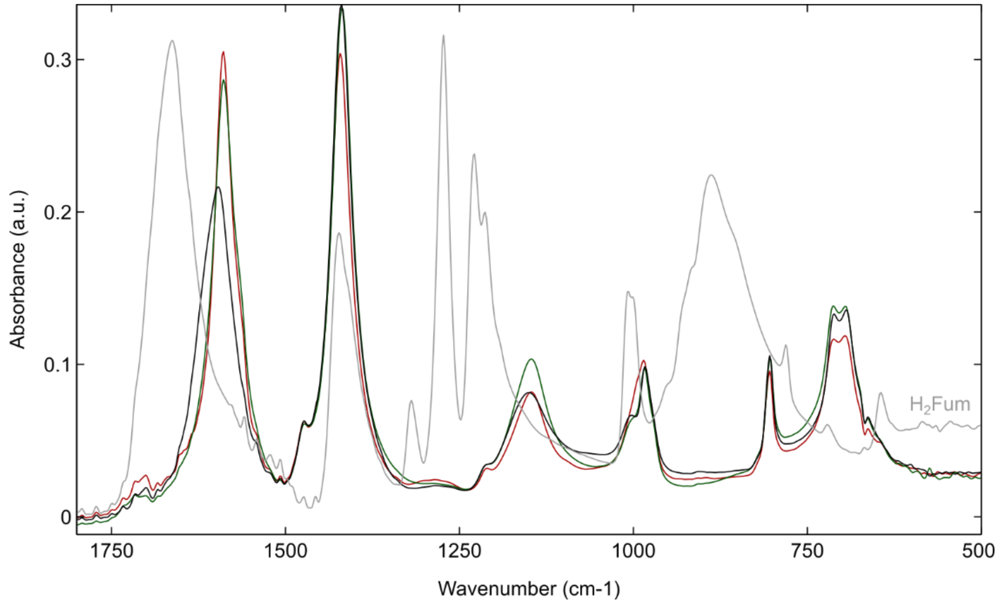

Figure S3.9 IR spectra of va-Al-MIL-53-Fum materials synthesized in presence and absence of formic acid vapour. No significant difference between Al-MIL-53-Fum synthesized in presence (green, entry 4 in Table S3.1) or absence of formic acid vapour (red, entry 8 in Table S3.1), or solvothermally following a reported procedure can be observed.[8] Also, the absence of the characteristic IR bands of the free (i.e. protonated) linker molecule (grey) in the Al-MIL-53 spectra confirms the proper activation of the materials.

S3.2. Vapour-Assisted Synthesis of Al-MIL-53-Mes and Mixed-Linker Al-MIL-53-Fum/Mes

Reaction conditions overview

For the vapour-assisted (va) syntheses of va-Al-MIL-53-Mes and mixed-linker Al-MIL-53-Fum/Mes, the conditions were identical to the optimised conditions for va-Al-MIL-53-Fum, only the fraction of fumaric acid and mesaconic acid in the reaction mixture was varied from 100% to 0% in 10% steps.

Table S3.2 Reaction details of the vapour-assisted synthesis optimisation of Al-MIL-53-Fum, Al-MIL-53-Mes and Mixed-Linker Al-MIL-53-Fum/Mes.

Al^{3+} source	Al^{3+}: H_2Fum : H_2Mes / molar	Additives[a]	Product
AlN	1:2:0	FA, Sat. KCl	va-Al-MIL-53-Fum
AlN	1:1.8:0.2	FA, Sat. KCl	va-Al-MIL-53-10%Mes
AlN	1:1.6:0.4	FA, Sat. KCl	va-Al-MIL-53-20%Mes
AlN	1:1.4:0.6	FA, Sat. KCl	va-Al-MIL-53-30%Mes
AlN	1:1.2:0.8	FA, Sat. KCl	va -Al-MIL-53-40%Mes
AlN	1:1:1	FA, Sat. KCl	va -Al-MIL-53-50%Mes
AlN	1:0.8:1.2	FA, Sat. KCl	va -Al-MIL-53-60%Mes
AlN	1:0.6:1.4	FA, Sat. KCl	va -Al-MIL-53-70%Mes
AlN	1:0.4:1.6	FA, Sat. KCl	va -Al-MIL-53-80%Mes
AlN	1:0.2:1.8	FA, Sat. KCl	va -Al-MIL-53-90%Mes
AlN	1:0:2	FA, Sat. KCl	va -Al-MIL-53-Mes

[a]formic acid (FA) or KCl saturated salt solution in water.

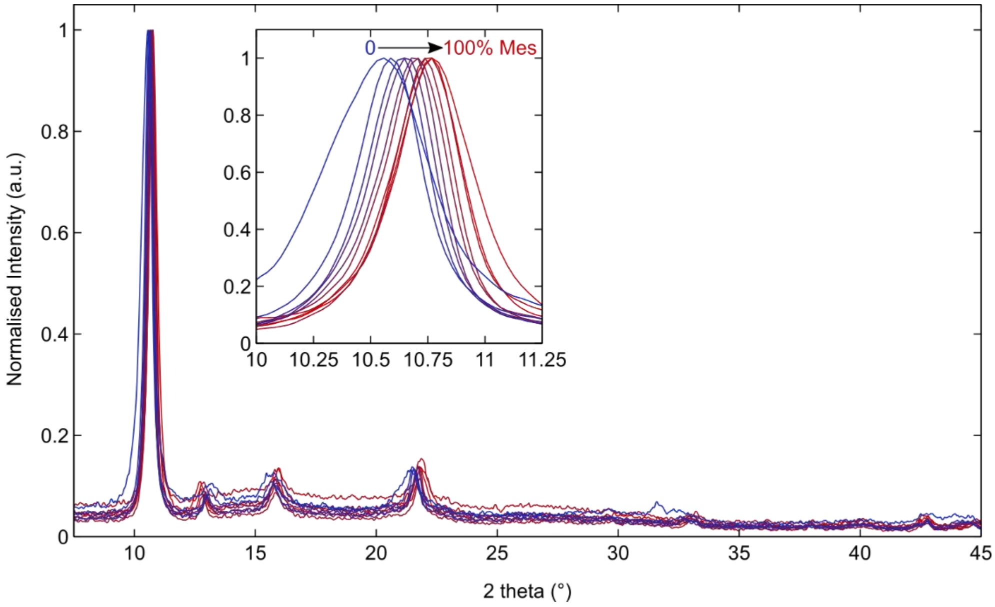

Figure S3.10 PXRD patterns of mixed-linker va-Al-MIL-53-Fum/Mes. Under the optimised reaction conditions for vapour-assisted synthesis and by simply varying the linker composition in the starting mixture between pure fumaric acid (blue) and pure mesaconic acid (red), mixed-linker Al-MIL-53-Fum/Mes can be prepared. All materials display the MIL-53 type structure as confirmed by PXRD. A gradual shift of the 011 reflection (inset) can be observed with increasing mesaconate content.

S4. Structure Refinement, Le Bail and Pawley Fits

Le Bail fit of fr-Al-MIL-53-Fum

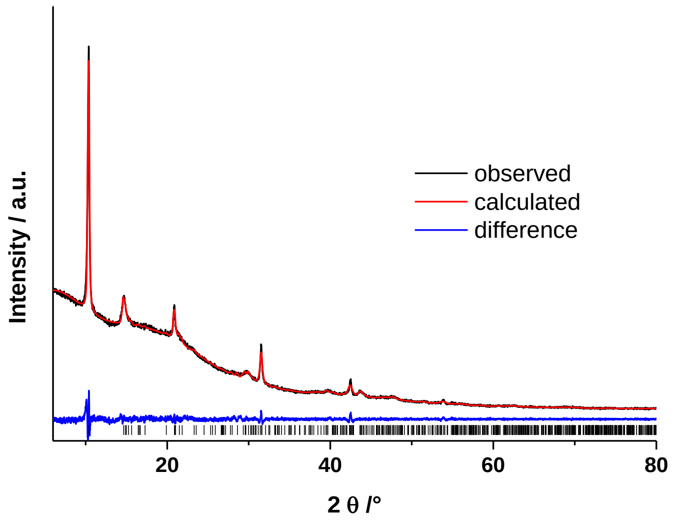

Figure S4.1 Le Bail fit of Al-MIL-53-Fum made in the flow reactor in the monoclinic space group $P2_1/c$, with the unit cell parameter a = 6.617(2) Å, b = 12.074(4) Å, c = 13.923(5) Å, β = 121.18(1)°. (R_{wp} = 2.7%, GOF = 1.2).

Le Bail fit of fr-Al-MIL-68-Mes

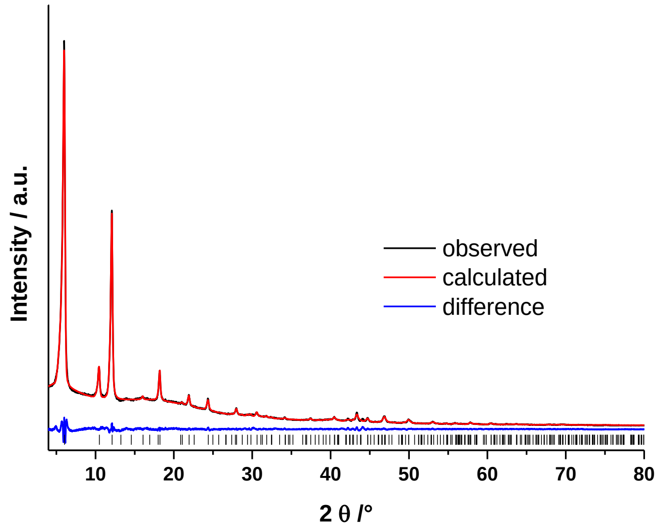

Figure S4.2 Le Bail fit of Al-MIL-68-Mes made in the flow reactor in the trigonal space group $P\overline{3}$ with the unit cell parameter a = 16.846(2) Å, c = 6.679(3) Å (R_{wp} = 3.4%, GOF = 1.8).

Structure refinement and crystallographic information for va-Al-MIL-53-Mes

Rietveld refinement

The PXRD pattern of va-Al-MIL-53-Mes was measured in transmission geometry on a STOE Stadi MP equipped with a Mythen detector and using Cu Kα_1 radiation. Ignoring two additional odd-shaped peaks (see below), it matches reasonably well with a primitive orthorhombic unit cell (a = 6.75, b = 14.34, c = 8.83 Å). To develop a structural model, the low symmetry (space group $P2_12_12_1$) crystal structure of Gallium camphorate with MIL-53 framework was used.[9] The metal atoms were changed, the camphorate linker molecules were replaced by mesaconate molecules (assuming full disorder of the methyl group) and after imposing the cell parameters, the model was optimized by force-field calculations using the universal force field as implemented in the Materials Studio software.[10] This resulted in a model with space group symmetry *Pnma*. The thus obtained model could be successfully refined by Rietveld methods with TOPAS,[11] using only distance restrains. In addition, a preferred orientation along [010] was considered and the peak broadening was modelled by 8th order spherical harmonics. Residual electron density inside the pores was modelled as oxygen atoms of refinable occupancy and one overall temperature factor was used. Some relevant parameters are summarized in Table S4.1 and the final plot is shown in Figure S4.3.

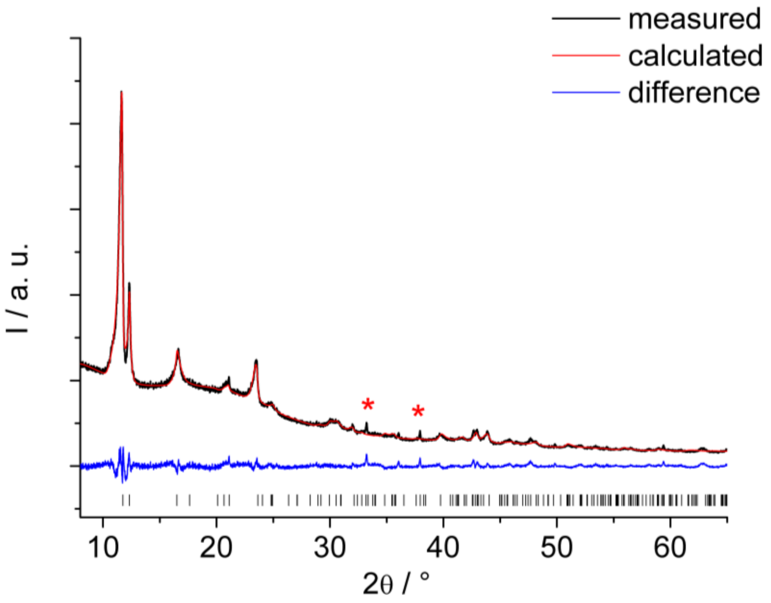

Figure S4.3 Plot for the Rietveld refinement of va-Al-MIL-53-Mes. The black curve represents the measured data, the red curve is the theoretical data and the blue curve indicates the difference. Vertical black bars mark the allowed Bragg reflection positions. The asterisks indicate a crystalline impurity.

Table S4.1 Relevant parameters for the Rietveld refinement of va-Al-MIL-53-Mes.

**Parameter**	**Value**
crystal system	orthorhombic
space group	*Pnma*
a / Å	6.756(4)
b / Å	14.341(4)
c / Å	8.832(2)
V/ Å^3	855.8(6)
R_{wp} / %	3.9
R_p / %	2.8
R_{Bragg} / %	0.7
GoF	2.9

Asymmetric unit and bond distances

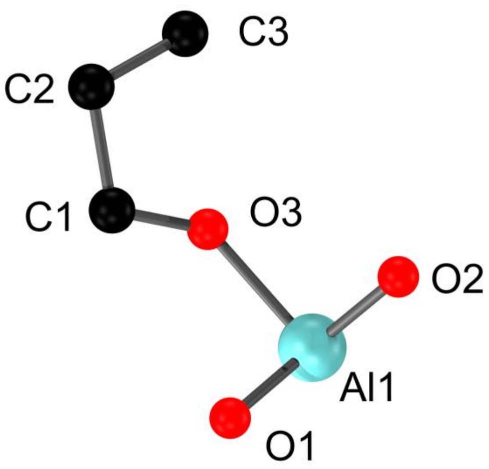

Figure S4.4 Asymmetric unit for va-Al-MIL-53-Mes with numbering scheme as used in Table S4.2. Guest atoms are omitted for clarity.

Table S4.2 Selected bond distances for Al-MIL-53-Mes.

**Atom**	**Atom**	**Bond distance**
Al1	O1	1.863(28)
	O2	1.909(15)
	O1	1.919(29)
	O3	1.938(15)
	C1	1.256(22)
O3	C2	1.542(20)
C1	C2	1.321(21)
C2	C3	1.504(27)

Crystallographic information file for Al-MIL-53-Mes

```
data_
_chemical_name_mineral 'Al-MIL-53-Mes'
_cell_length_a  6.7558(43)
_cell_length_b  14.3407(38)
_cell_length_c  8.8329(25)
_cell_angle_alpha 90
_cell_angle_beta  90
_cell_angle_gamma 90
_cell_volume 855.76(64)
_symmetry_space_group_name_H-M PNMA
loop_
_symmetry_equiv_pos_as_xyz
	'-x, -y, -z'
	'-x, y+1/2, -z'
	'-x+1/2, -y, z+1/2'
	'-x+1/2, y+1/2, z+1/2'
	'x, -y+1/2, z'
	'x, y, z'
	'x+1/2, -y+1/2, -z+1/2'
	'x+1/2, y, -z+1/2'
loop_
_atom_site_label
_atom_site_type_symbol
_atom_site_symmetry_multiplicity
_atom_site_fract_x
_atom_site_fract_y
_atom_site_fract_z
_atom_site_occupancy
_atom_site_B_iso_or_equiv
Al1 Al   0 0.1976(24) 0.25 0.2435(15) 1 0.51(42)
O1 O   0 0.4565(38) 0.25 0.1541(15) 1 0.51(42)
O2 O   0 0.1188(14) 0.34470(73) 0.1040(13) 1 0.51(42)
O3 O   0 0.2901(13) 0.34479(80) 0.3829(14) 1 0.51(42)
C1 C   0 0.4493(31) 0.37694(76) 0.4346(21) 1 0.51(42)
C2 C   0 0.4189(24) 0.47444(71) 0.5045(34) 1 0.51(42)
C3 C   0 0.1982(29) 0.4842(37) 0.5200(86) 0.5 0.51(42)
Ow1 O   0 -0.022(14) 0.7246(31) 0.3193(43) 0.362(17) 0.51(42)
```

Pawley fit of va-Al-MIL-53-Fum

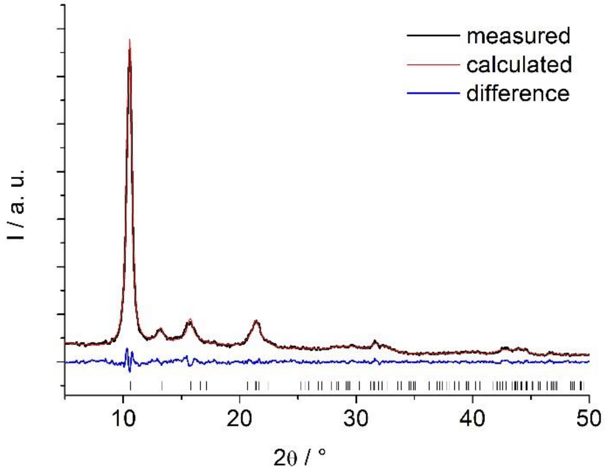

Figure S4.5 Pawley fit of va-Al-MIL-53-Fum in the orthorhombic space group *Pnma* with the unit cell parameter a = 6.583(5), b = 13.312(12) and c = 10.674(7).

S5. ^{1}H NMR Spectroscopy

In order to measure ^{1}H NMR spectra, the samples made in the flow reactor were dissolved in a NaOD/D_2O mixture, and the samples made via vapour-assisted synthesis were dissolved in a DF/D_2O/DMSO-d_6 mixture. For a better overview only the results from fr-Al-MIL-53-45%Mes are displayed graphically (Figure S5.1). All other NMR spectra were processed the same way and the results are summarized in Tables S5.1 and S5.2.

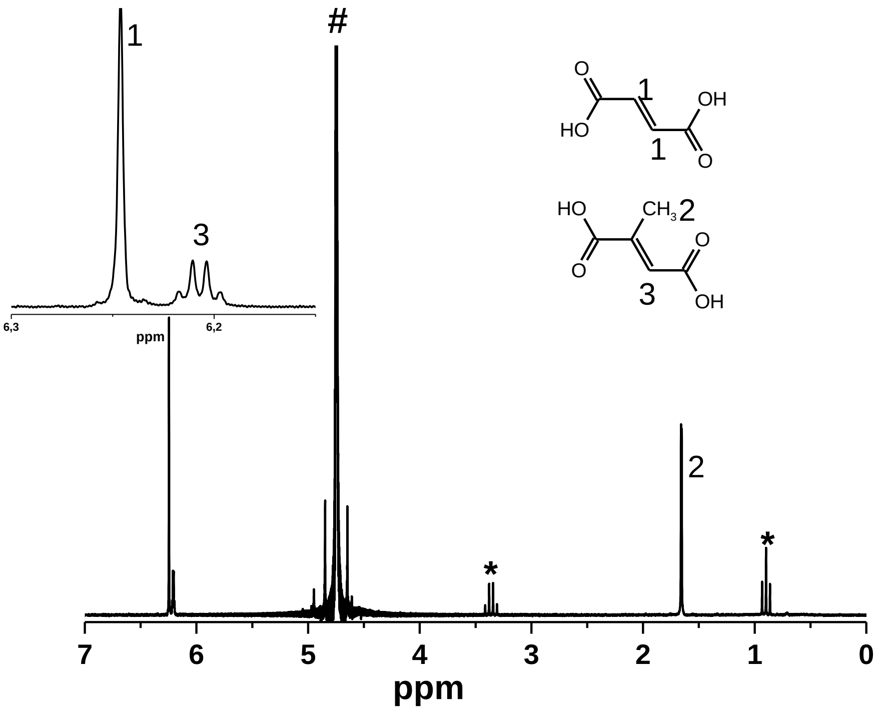

Figure S5.1 ^{1}H NMR-spectroscopy of fr-Al-MIL-53-45%Mes dissolved in a NaOD/D_2O mixture. The star indicates proton signals from ethanol which was used for washing the samples prepared in the flow reactor. The numbers indicate the protons from the linker molecules used for quantification.

Table S5.1 Incorporated mesaconate content in the materials synthesized in the flow reactor quantified by ^{1}H NMR-spectroscopy.

**Material**	**Mes^{2-} fraction in precursor solution/ %**	**Incorporated. Mes^{2-} / %**	
		Replicate. 1	Replicate. 2
fr-Al-MIL-68-Mes	100	100	100
fr-Al-MIL-68-90%Mes	90	86	90
fr-Al-MIL-68-80%Mes	80	83	81
fr-Al-MIL-68-70%Mes	70	67	70
fr-Al-MIL-68-60%Mes	60	63	63
fr-Al-MIL-68-55%Mes	55	58	57
fr-Al-MIL-53-45%Mes	45	46	46
fr-Al-MIL-53-40%Mes	40	38	39
fr-Al-MIL-53-30%Mes	30	32	30
fr-Al-MIL-53-20%Mes	20	21	19
fr-Al-MIL-53-10%Mes	10	10	10
fr-Al-MIL-53-Fum	0	0	0

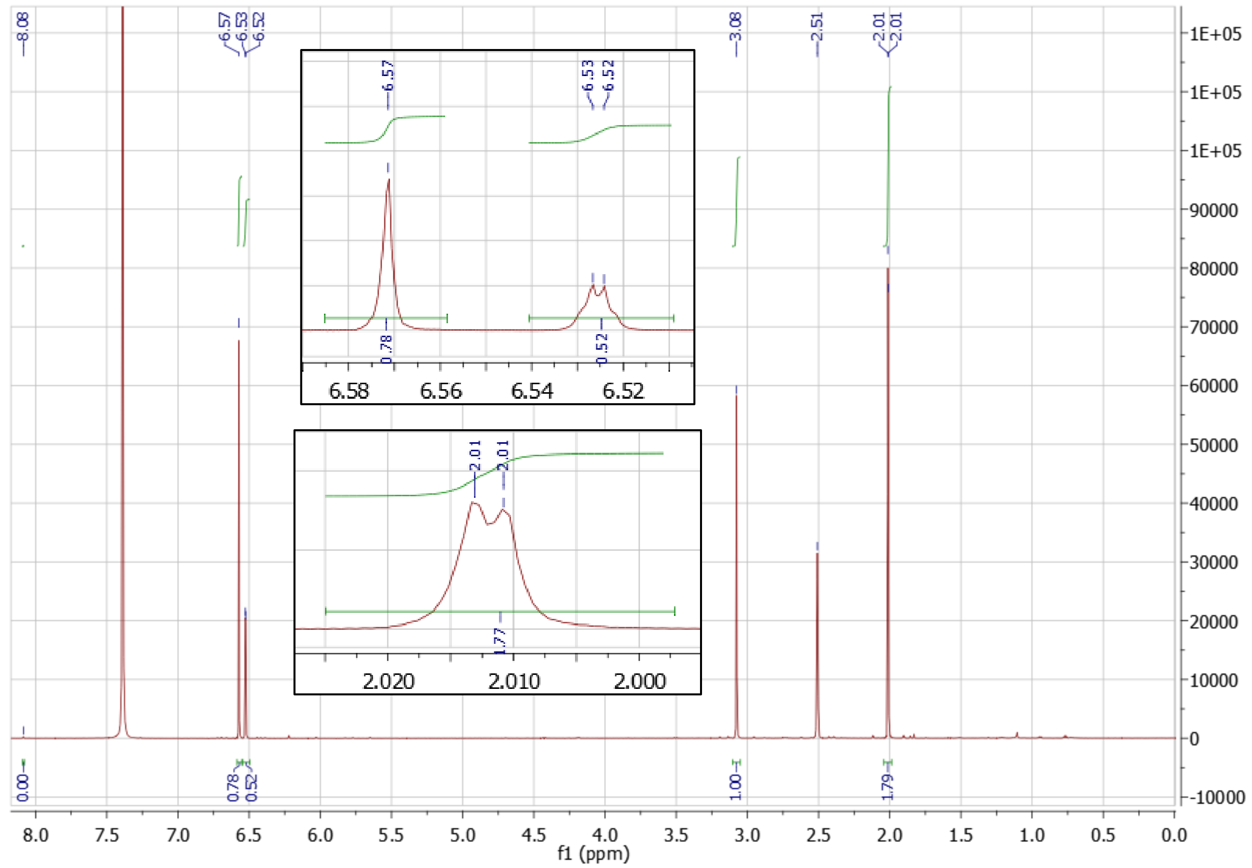

Figure S5.2 ^{1}H NMR-spectroscopy of va-Al-MIL-53-60%Mes dissolved in a DF/D_2O/DMSO-d_6 mixture evidencing the absence of formate ions (δ 8.08) in the final material: ^{1}H NMR(DF/D_2O/DMSO-d_6) δ 2.015 (d, 3H, HOOC-CH=CC**H**$_3$-COOH), δ 6.525 (q, 1H, HOOC-C**H**=CCH$_3$-COOH), δ 6.57 (s, 2H, HOOC-CH=C**H**-COOH).

Table S5.2 Incorporated mesaconate content in the materials prepared via vapour-assisted synthesis quantified by ^{1}H NMR-spectroscopy.

**Material**	**Mes^{2-} fraction in precursor powder mixture / %**	**Incorporated Mes^{2-} / %** Replicate. 1	Replicate. 2
va-Al-MIL-53-Mes	100	0	0
va-Al-MIL-53-90%Mes	90	82	81
va-Al-MIL-53-80%Mes	80	75	74
va-Al-MIL-53-70%Mes	70	65	68
va-Al-MIL-53-60%Mes	60	60	62
va-Al-MIL-53-50%Mes	50	56	56
va-Al-MIL-53-40%Mes	40	48	49
va-Al-MIL-53-30%Mes	30	40	40
va-Al-MIL-53-20%Mes	20	24	28
va-Al-MIL-53-10%Mes	10	14	15
va-Al-MIL-53-Fum	0	0	0

S6. Elemental Analyses

Table S6.1 Elemental analysis of fr-Al-MIL-68-Mes, fr-Al-MIL-53-Fum and the mixed-linker products compared to the expected values for pure Al-MIL-53-Mes ([Al(OH)(Mes)]) and Al-MIL-53-Fum ([Al(OH)(Fum)]). Most of the samples show a small amount of sulphur (from the aluminium sulfate precursor salt), which explains the difference from the calculated and measured values of carbon and hydrogen. However, the measured elemental fractions appear to be reproducible.

Material	C %	Rep. C %	H %	Rep. H %	S %	Rep. S %
**Theo. Al-MIL-68-Mes**	34.3	---	4.6	---	0	---
**fr-Al-MIL-68-Mes**	31.7	---	2.9	---	0.9	---
**fr-Al-MIL-68-90%Mes**	32.3	30.2	2.7	2.7	0.8	1.6
**fr-Al-MIL-68-80%Mes**	28.2	28.4	2.9	2.4	0	0
**fr-Al-MIL-68-70%Mes**	27.8	29.9	2.2	2.3	1.3	0.6
**fr-Al-MIL-68-60%Mes**	31.1	32.2	2.9	2.7	0	0
**fr-Al-MIL-68-55%Mes**	30.0	27.7	2.5	2.3	0	0.2
**fr-Al-MIL-53-45%Mes**	27.9	30.4	2.5	2.8	2.4	1.0
**fr-Al-MIL-53-40%Mes**	26.3	26.4	2.3	2.5	1.8	1.9
**fr-Al-MIL-53-30%Mes**	26.0	29.3	2.1	2.4	1.1	1.4
**fr-Al-MIL-53-20%Mes**	29.6	29.4	3.4	3.1	1.3	1.3
**fr-Al-MIL-53-10%Mes**	27.4	27.4	2.2	2.6	1.8	1.4
**fr_MIL-53-Fum**	27.8	---	2.1	---	1.6	---
**Theo. Al-MIL-53-Fum**	30.2	---	2.5	---	0	---

Table S6.2 Elemental analysis of va-Al-MIL-53-Mes, va-Al-MIL-53-Fum and the mixed-linker va-products obtained compared to the expected values for pure Al-MIL-53-Mes ([Al(OH)(Mes)]) and Al-MIL-53-Fum ([Al(OH)(Fum)]). The much larger amount of nitrogen observed in the sample prepared in absence of formic acid vapour (highlighted with a red box) confirms the incomplete aluminium nitride hydrolysis and conversion to the MOF. Otherwise the measured elemental fractions appear to be reproducible.

Material	C %	Rep. C %	H %	Rep. H %	N %	Rep. N %
**Theo. Al-MIL-68-Mes**	34.3	34.3	4.6	4.6	0	0
**va-Al-MIL-53-Mes**	26.3	32.3	4.1	3.7	0.6	0.3
**va-Al-MIL-53-90%Mes**	32.4	32.7	3.8	3.5	0.2	0.5
**va-Al-MIL-53-80%Mes**	32.7	30.1	3.7	3.2	0.4	0.4
**va-Al-MIL-53-70%Mes**	28.9	27.5	2.9	2.9	0.7	0.6
**va-Al-MIL-53-60%Mes**	37.5	30.6	3.7	3.2	0.9	1.2
**va-Al-MIL-53-50%Mes**	30.0	30.5	4.3	3.1	0.7	0.7
**va-Al-MIL-53-40%Mes**	30.7	30.5	4.8	3.2	0.5	0.6
**va-Al-MIL-53-30%Mes**	25.6	29.9	4.2	3.3	0.5	0.6
**va-Al-MIL-53-20%Mes**	26.7	27.7	4.1	3.8	0.5	0.4
**va-Al-MIL-53-10%Mes**	23.4	27.1	4.3	3.8	0.3	0.4
**va-Al-MIL-53-Fum**	35.6	25.1	3.5	4.1	1.0	0.5
**va-Al-MIL-53-Fum (no FA)**	---	18.2	---	3.4	---	7.6
**Theo. Al-MIL-53-Fum**	30.2	30.2	2.5	2.5	0	0

S7. Thermogravimetric Analyses

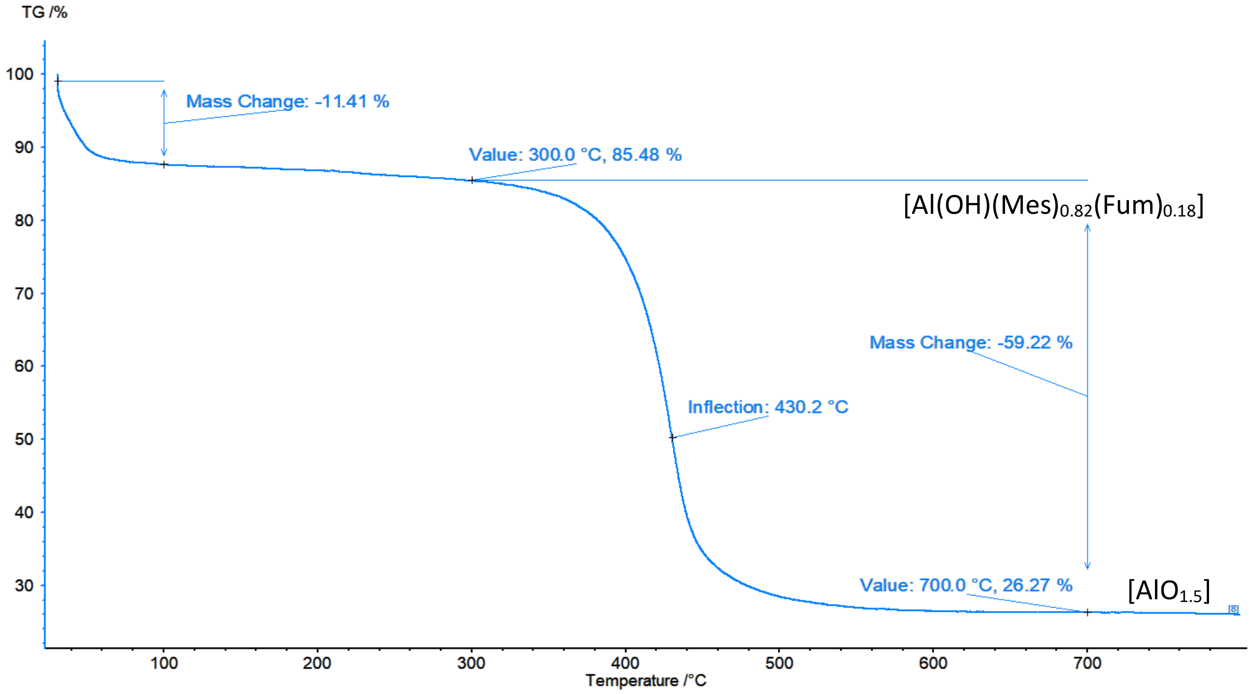

Figure S7.1 Exemplary TGA plot for fr-Al-MIL-68-80%Mes. The weight loss at low temperatures is attributed to pore degassing. The theoretical wt% linker can be calculated as wt% = 1-[$AlO_{1.5}$]/[Al(OH)$(Mes)_{0.82}(Fum)_{0.18}$] = 1–51/169.5 = 70%. The measured wt% linker is wt% = 1-[$wt\%_{700°C}$]/ [$wt\%_{300°C}$] = 1–26.27/85.48 = 69%.

Table S7.1 Results of the thermogravimetric analysis of fr-Al-MIL-68-Mes, fr-Al-MIL-53-Fum and the mixed-linker products compared to the theoretical values calculated for the formula [Al(OH)$(Mes)_x(Fum)_{1-x}$]. The values of x were calculated from ^{1}H NMR-spectroscopy data.

Material	Theoretical wt% linker	Measured wt% linker	Decomposition Temperature[a] / °C
**fr-Al-MIL-68-Mes**	70	64	425.5
**fr-Al-MIL-68-90%Mes**	70	-	-
**fr-Al-MIL-68-80%Mes**	70	69	430.2
**fr-Al-MIL-68-70%Mes**	70	67	432.1
**fr-Al-MIL-68-60%Mes**	69	50	465.1
**fr-Al-MIL-68-55%Mes**	69	69	439.3
**fr-Al-MIL-53-45%Mes**	69	61	428.1
**fr-Al-MIL-53-40%Mes**	69	62	433.2
**fr-Al-MIL-53-30%Mes**	69	58	422.9
**fr-Al-MIL-53-20%Mes**	68	63	447.8
**fr-Al-MIL-53-10%Mes**	68	50	454.6
**fr-Al-MIL-53-Fum**	68	59	445.3

[a]considered here as the inflection point of the TG trace at high temperatures.

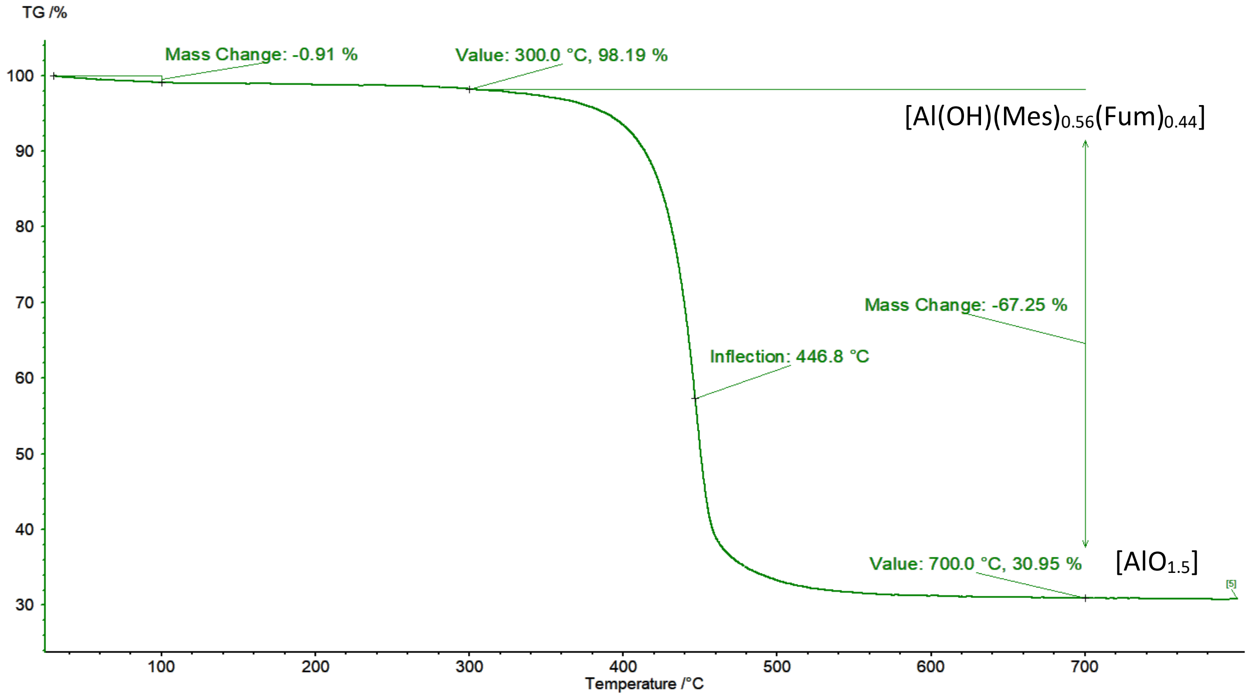

Figure S7.2 Exemplary TGA plot for va-Al-MIL-53-50%Mes. The weight loss at low temperatures is attributed to pore degassing. The theoretical wt% linker can be calculated as wt% = 1-[$AlO_{1.5}$]/[$Al(OH)(Mes)_{0.82}(Fum)_{0.18}$] = 1–51/165.4 = 69%. The measured wt% linker is wt% = 1-[$wt\%_{700°C}$]/ [$wt\%_{300°C}$] = 1–30.95/98.19 = 68%. From the results of the elemental analyses, the absence of aluminium nitride can safely be assumed.

Table S7.2 Results of the thermogravimetric analysis of va-Al-MIL-68-Mes, va-Al-MIL-53-Fum and the mixed-linker va-products compared to the theoretical values calculated for the formula [$Al(OH)(Mes)_x(Fum)_{1-x}$]. The values of x were calculated from ^1H NMR-spectroscopy data.

Material	Theoretical wt% linker	Measured wt% linker	Decomposition Temperature^a / °C
**va-Al-MIL-53-Mes**	70%	71%	393.9
**va-Al-MIL-53-90%Mes**	70%	71%	415.1
**va-Al-MIL-53-80%Mes**	70%	69%	435.8
**va-Al-MIL-53-70%Mes**	69%	69%	443.4
**va-Al-MIL-53-60%Mes**	69%	69%	443.9
**va-Al-MIL-53-50%Mes**	69%	68%	446.8
**va-Al-MIL-53-40%Mes**	69%	69%	451.3
**va-Al-MIL-53-30%Mes**	69%	69%	455.5
**va-Al-MIL-53-20%Mes**	68%	69%	461
**va-Al-MIL-53-10%Mes**	68%	68%	465.9
**va-Al-MIL-53-Fum**	68%	68%	468.2

^{a}considered here as the inflection point of the TG trace at high temperatures.

S8. Fourier-Transform Infrared Spectroscopy

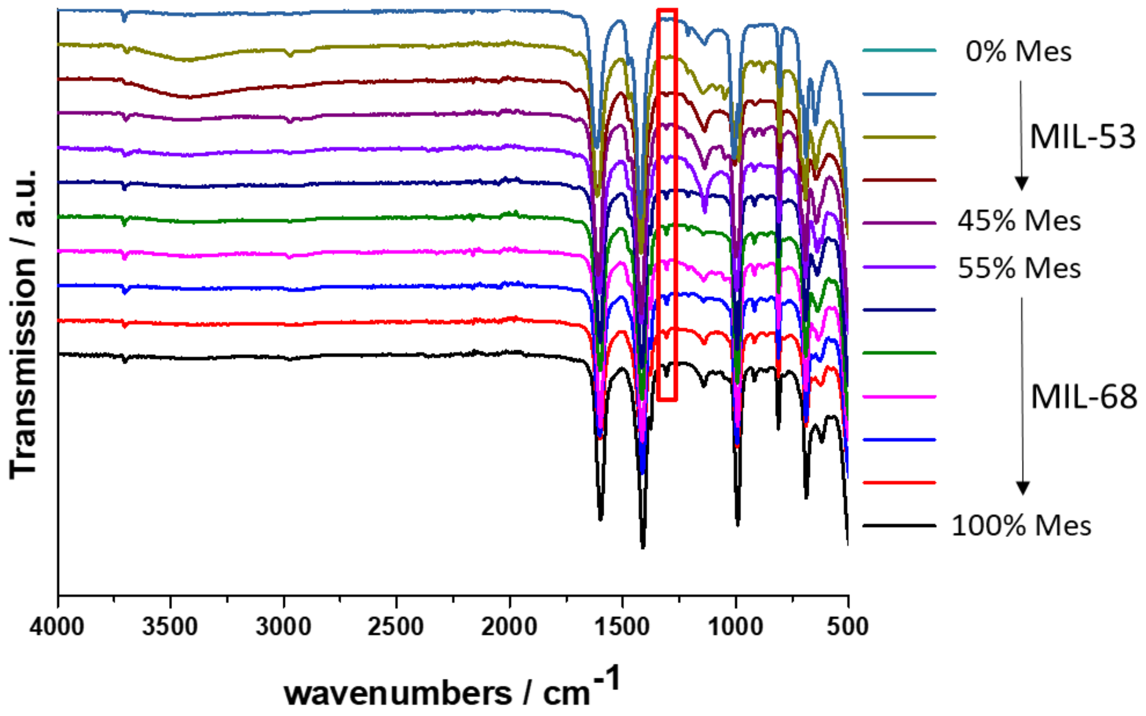

Figure S8.1 FTIR spectra of fr-Al-MIL-68-Mes (100% Mes), fr-Al-MIL-53-Fum (0% Mes) and all mixed-linker products in between. The red box highlights the increasing signal for the symmetric CH_3-deformation vibration for the increasing amount of Mes^{2-} ions in the samples.

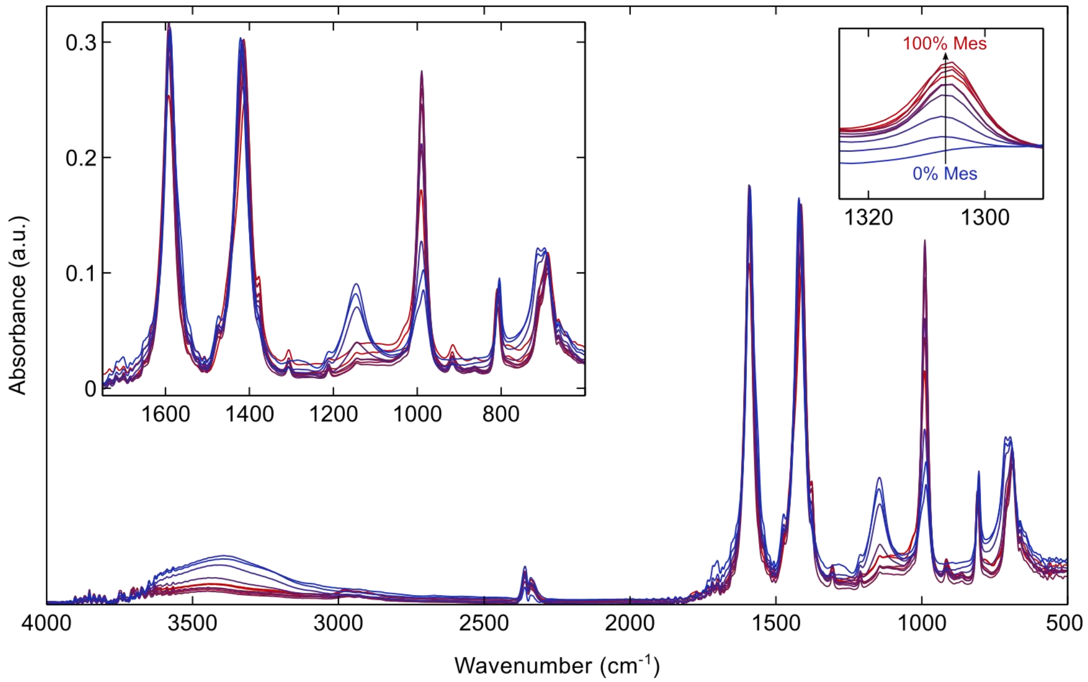

Figure S8.2 FTIR spectra of va-Al-MIL-53-Mes (100% Mes), va-Al-MIL-53-Fum (0% Mes) and all mixed-linker products in between. The left inset shows a zoom of the 600-1750 cm^{-1} range while the upper right inset highlights the increasing signal for the symmetric CH_3-deformation vibration with increasing amount of Mes^{2-} in the materials.

Table S8.1 FTIR spectra bands assignment.

Material	Wavenumber / cm^{-1}	Intensity	Classification
**fr-Al-MIL-68-Mes**	3702	w	OH stretch
	1600	s	Asymmetric CO_2 stretch
	1409	s	Symmetric CO_2 stretch
	1310	w	Symmetric CH_3 deformation
**fr-Al-MIL-53-Fum**	3702	w	OH stretch
	1616	s	Asymmetric CO_2 stretch
	1422	s	Symmetric CO_2 stretch
**va-Al-MIL-53-Mes**	1593	s	Asymmetric CO_2 stretch
	1412	s	Symmetric CO_2 stretch
	1306	w	Symmetric CH_3 deformation
**va-Al-MIL-53-Fum**	1589	s	Asymmetric CO_2 stretch
	1421	s	Symmetric CO_2 stretch

S9. Scanning Electron Microscopy

fr-Al-MIL-68-Mes

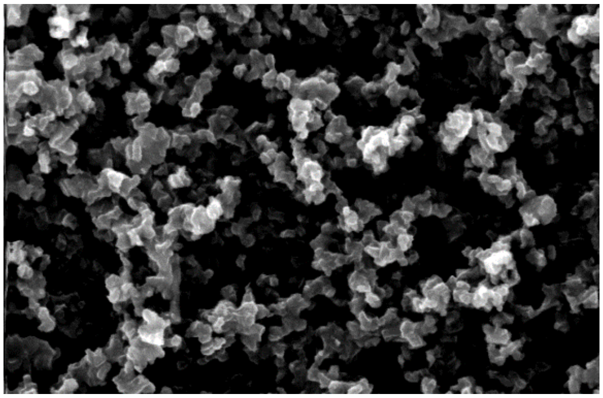

fr-Al-MIL-68-10%Mes

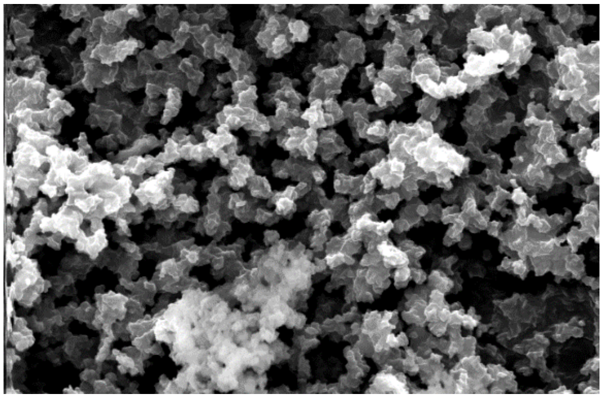

fr-Al-MIL-68-20%Mes

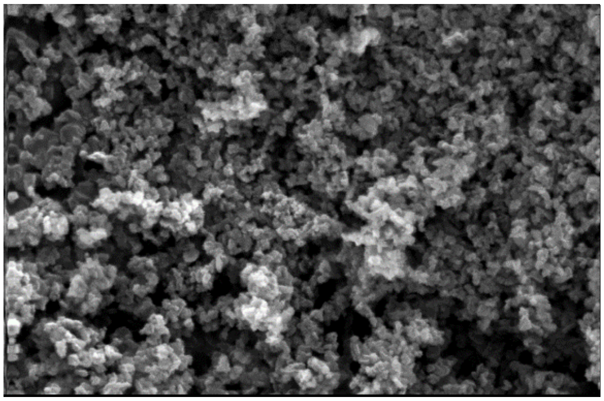

fr-Al-MIL-68-30%Mes

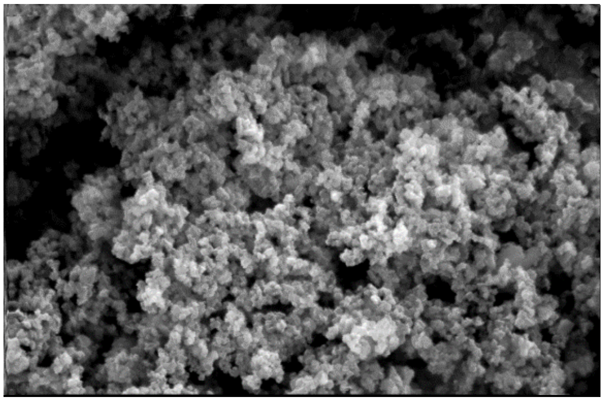

fr-Al-MIL-68-40%Mes

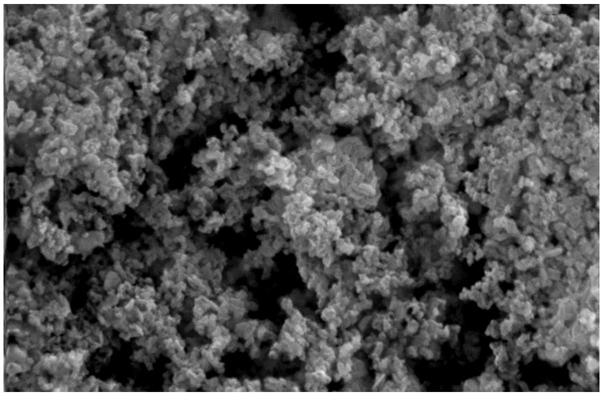

fr-Al-MIL-68-45%Mes

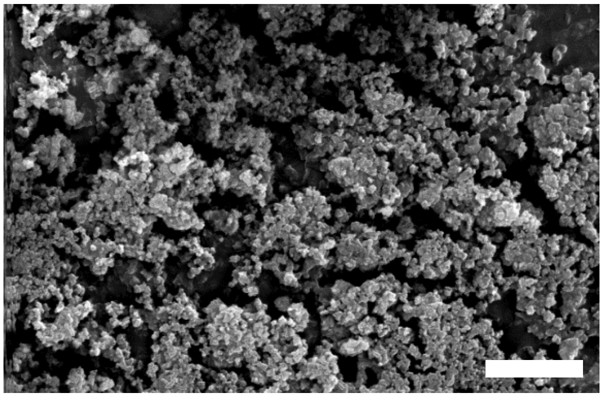

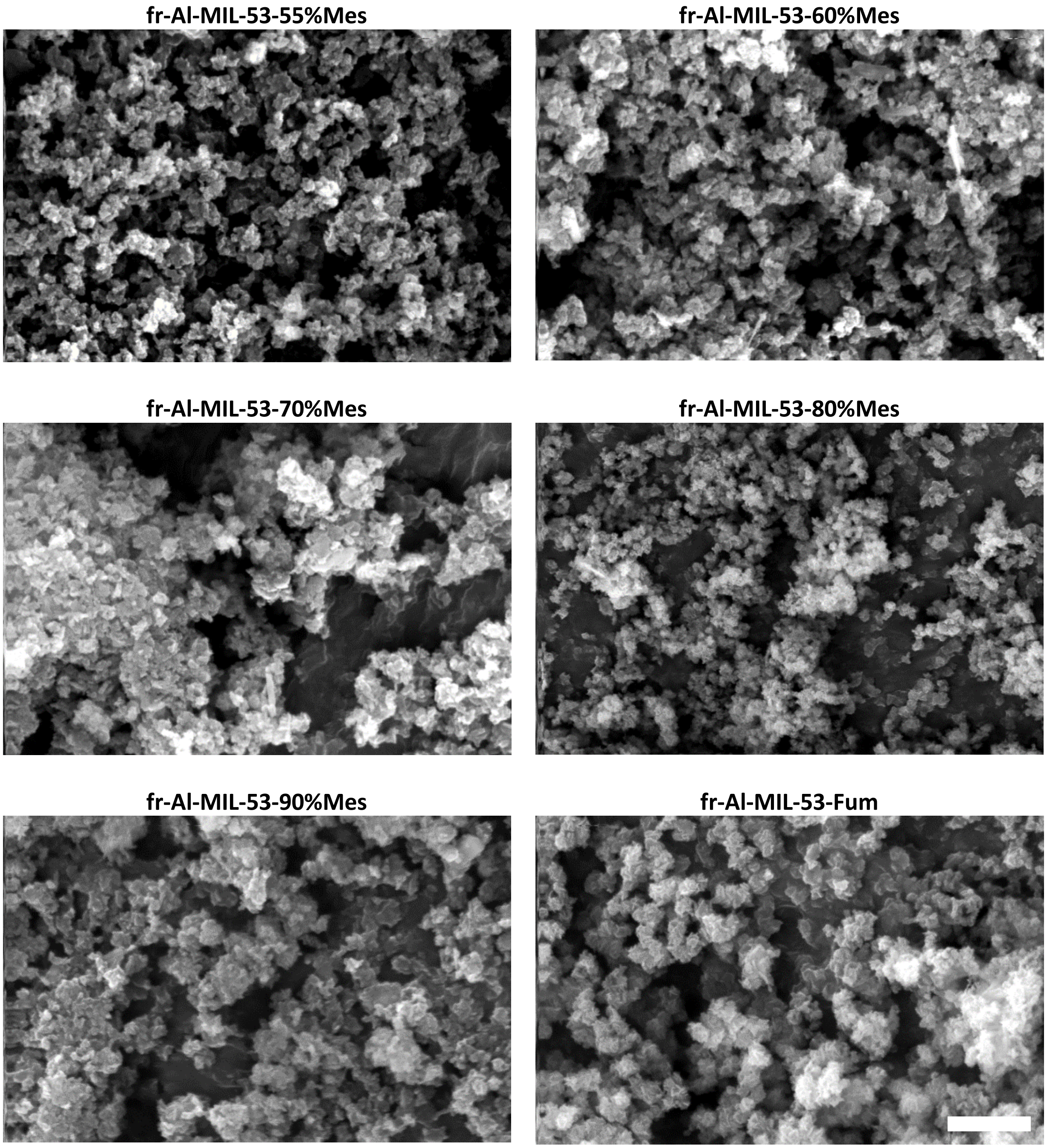

Figure S9.1 SEM micrographs of fr-Al-MIL-68-Mes, fr-Al-MIL-53-Fum and all mixed-linker products in between. The crystallites have no specific morphology and are smaller than 1 µm. Scale bar = 1 µm.

Va-Al-MIL-53-Fum - No FA

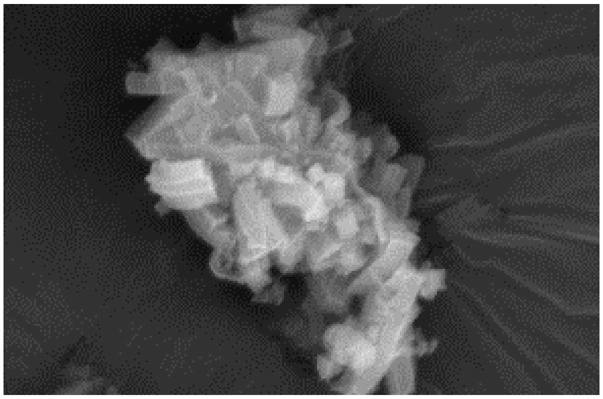

Va-Al-MIL-53-Fum - No FA

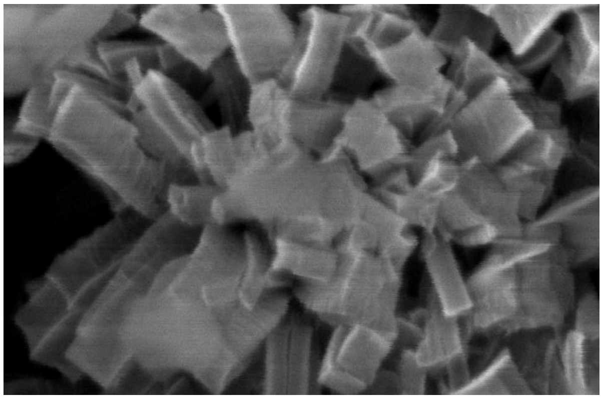

va-Al-MIL-53-Fum

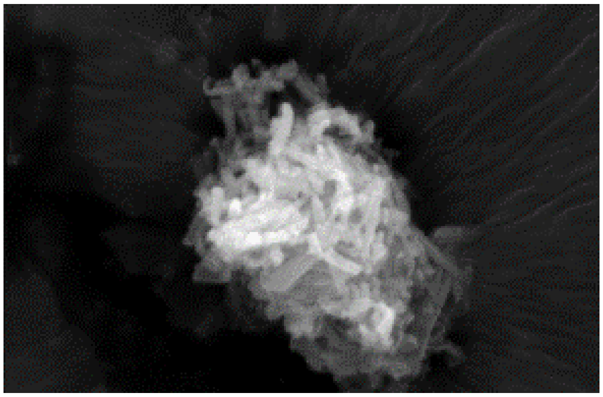

va-Al-MIL-53-Fum

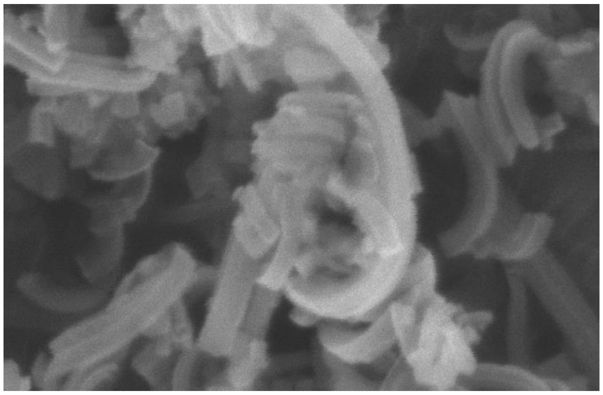

va-Al-MIL-53-10%Mes

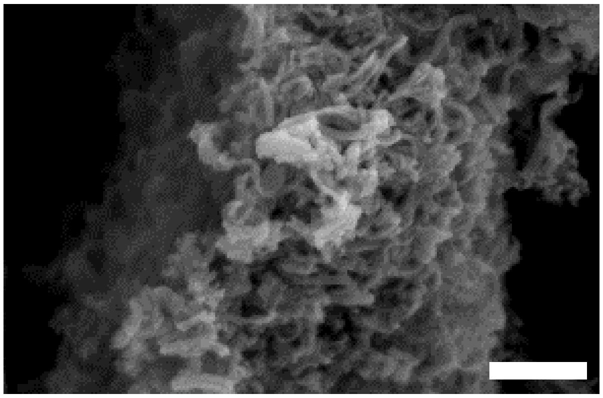

va-Al-MIL-53-10%Mes

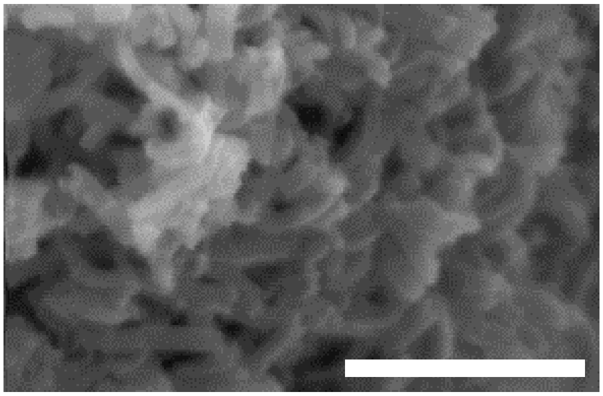

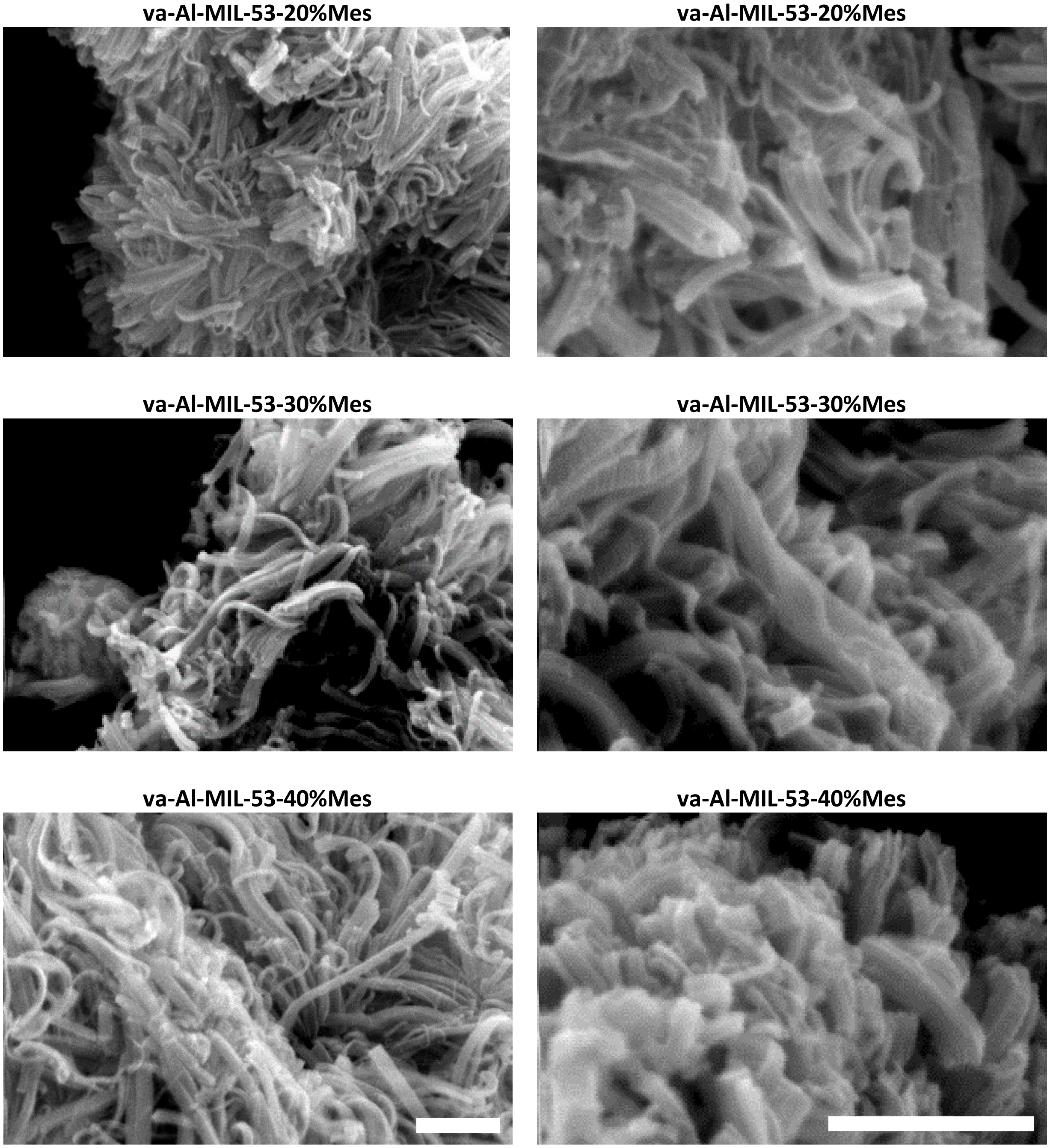
va-Al-MIL-53-20%Mes
va-Al-MIL-53-20%Mes
va-Al-MIL-53-30%Mes
va-Al-MIL-53-30%Mes
va-Al-MIL-53-40%Mes
va-Al-MIL-53-40%Mes

va-Al-MIL-53-50%Mes

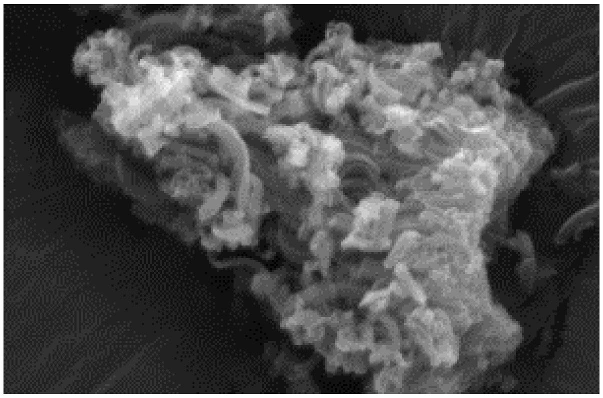

va-Al-MIL-53-50%Mes

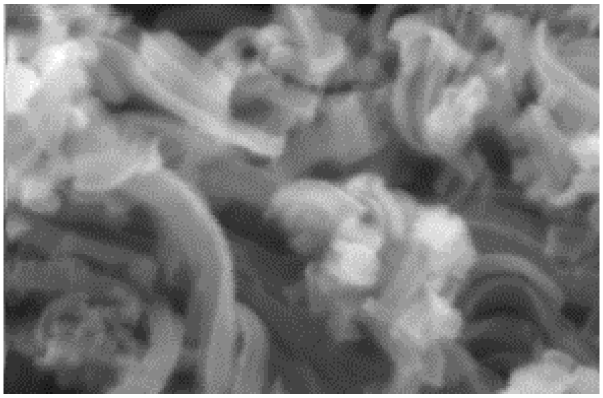

va-Al-MIL-53-60%Mes

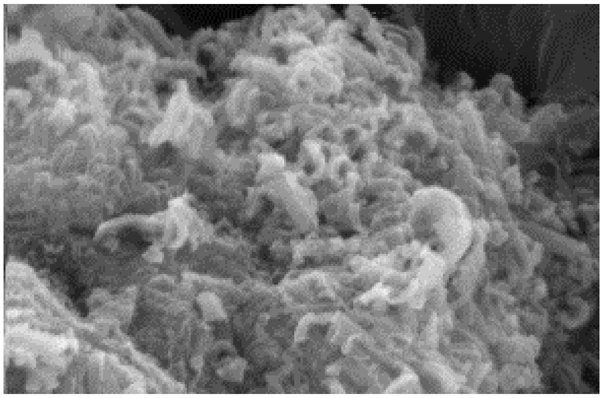

va-Al-MIL-53-60%Mes

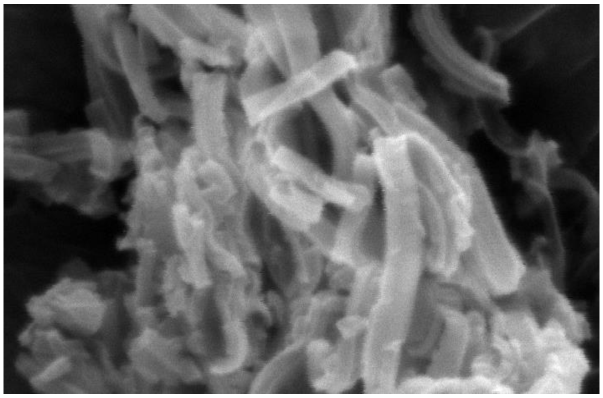

va-Al-MIL-53-70%Mes

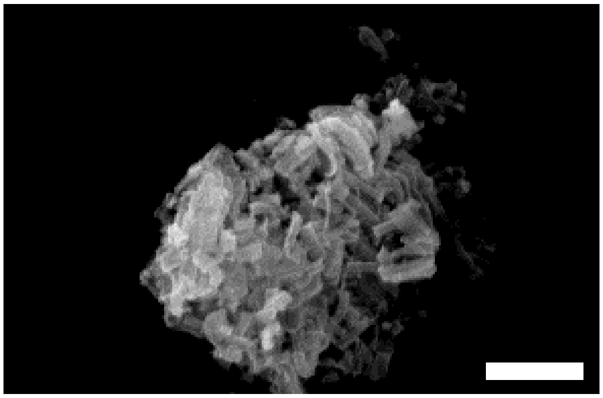

va-Al-MIL-53-70%Mes

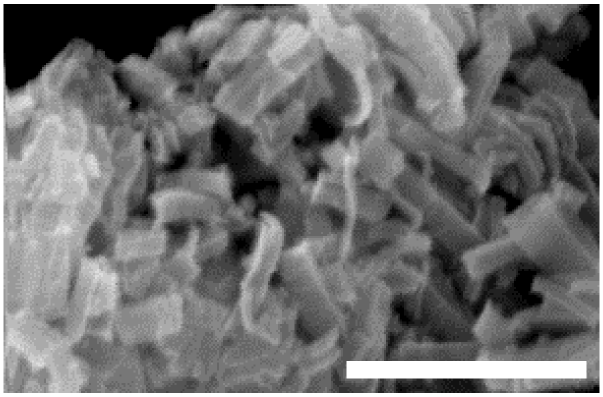

va-Al-MIL-53-80%Mes

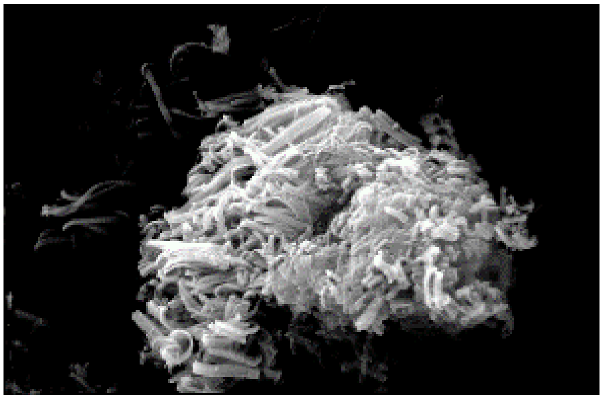

va-Al-MIL-53-80%Mes

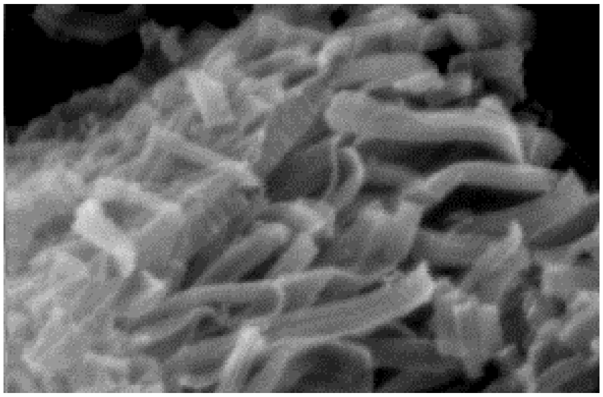

va-Al-MIL-53-90%Mes

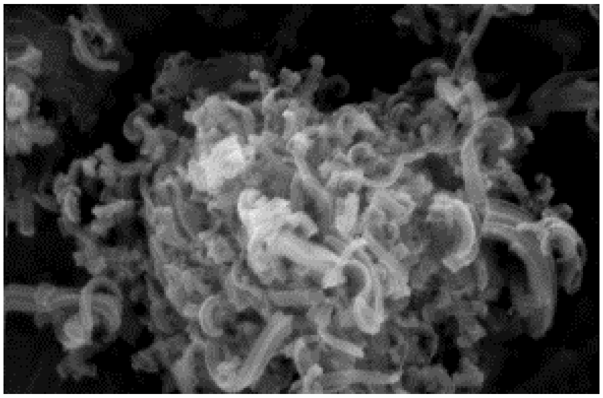

va-Al-MIL-53-90%Mes

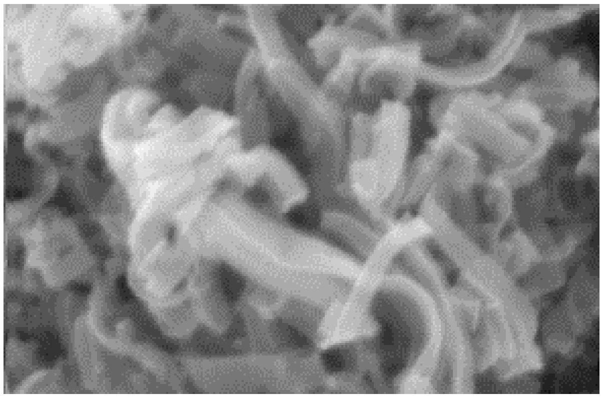

va-Al-MIL-53-Mes

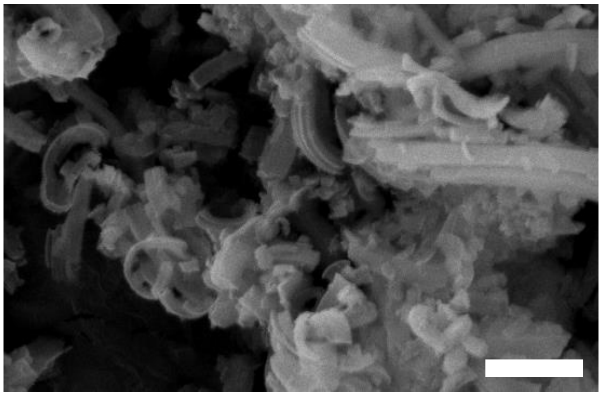

va-Al-MIL-53-Mes

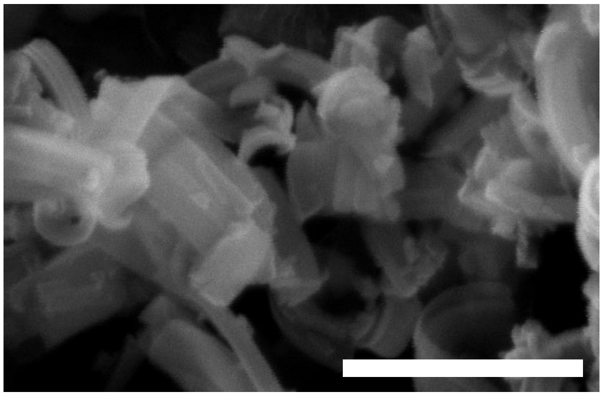

Figure S9.2 SEM micrographs of va-Al-MIL-53-Mes, va-Al-MIL-53-Fum and all mixed-linker products in between, as well as va-Al-MIL-53-Fum prepared in absence of formic acid vapour (entry 4 in Table S3.1). Scale bars = 1 µm.

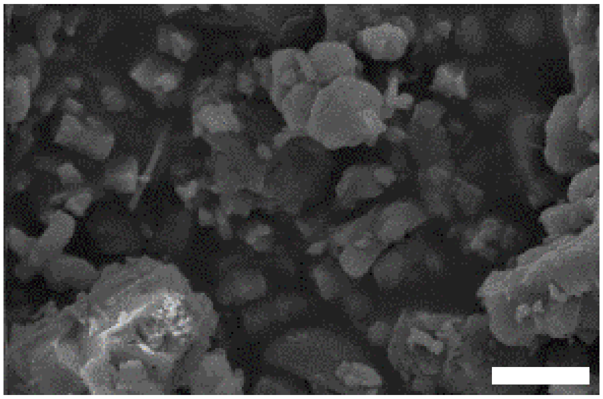

Figure S9.3 SEM micrographs of the aluminium nitride precursor powder. Scale bar = 1 µm.

S10. Nitrogen Physisorption

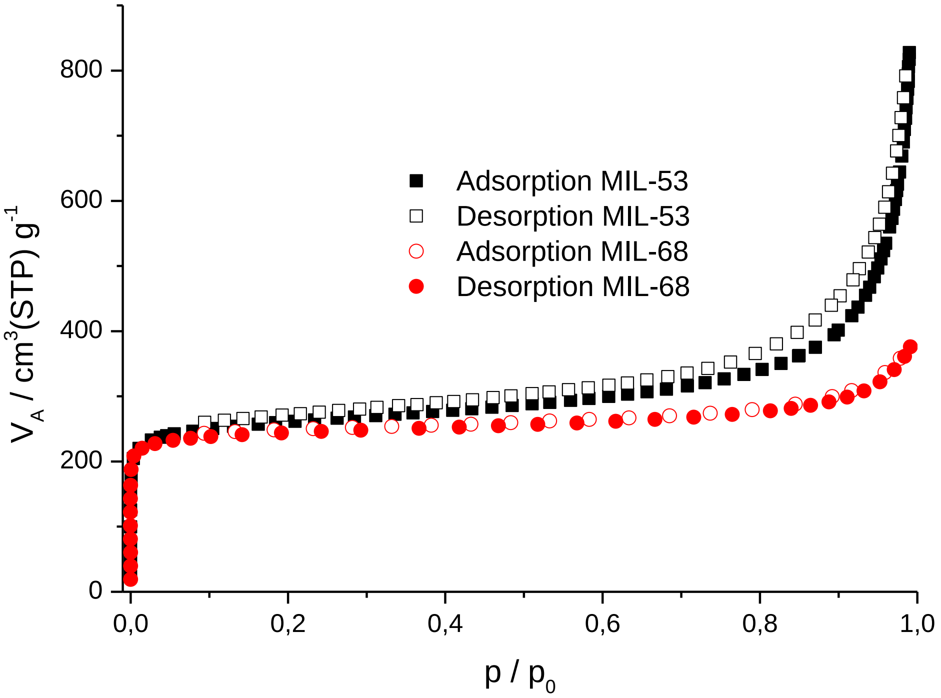

Figure S10.1 N_2 adsorption (filled) and desorption (empty) isotherm of fr-Al-MIL-53-Fum (black squares) and fr-Al-MIL-68-Mes (red circle) measured at -196 °C. The type 1 shape of both isotherms is characteristic for microporous materials.

Table S10.1 Specific surface area and micropore volume extracted from N_2 physisorption data for the materials prepared with the flow reactor and their replicates.

Material	$a_{s,BET}$* / $m^2 \cdot g^{-1}$		$V_{mic.}$# / $cm^3 \cdot g^{-1}$	
**fr-Al-MIL-68-Mes**	950	---	0.44	---
**fr-Al-MIL-68-90%Mes**	1080	1050	0.47	0.46
**fr-Al-MIL-68-80%Mes**	1260	1230	0.53	0.51
**fr-Al-MIL-68-70%Mes**	1090	1090	0.47	0.47
**fr-Al-MIL-68-60%Mes**	1360	1380	0.54	0.55
**fr-Al-MIL-68-55%Mes**	1070	1020	0.49	0.46
**fr-Al-MIL-53-45%Mes**	910	880	0.42	0.41
**fr-Al-MIL-53-40%Mes**	930	920	0.42	0.40
**fr-Al-MIL-53-30%Mes**	870	870	0.39	0.40
**fr-Al-MIL-53-20%Mes**	990	950	0.45	0.45
**fr-Al-MIL-53-10%Mes**	1000	960	0.45	0.44
**fr-Al-MIL-53-Fum**	1000	---	0.44	---

*The specific surface area was calculated following the Rouquerol criteria.[7] #The micropore volume was calculated at $P/P_0 = 0.5$.

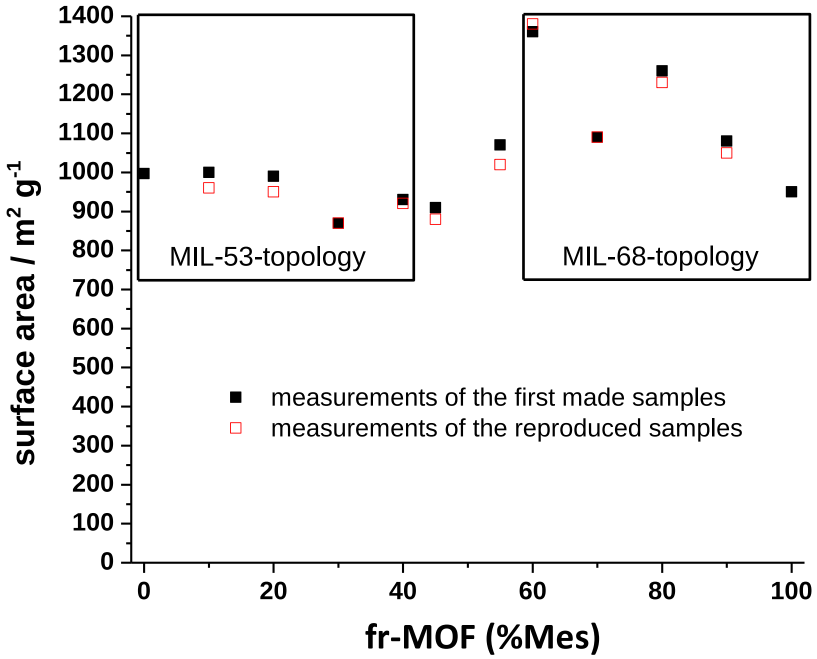

Figure S10.2 Specific surface area as a function of mesaconate content for the materials prepared with the flow reactor (full squares) and their replicates (empty squares). The boxes distinguishes the two corresponding framework topologies which are obtained.

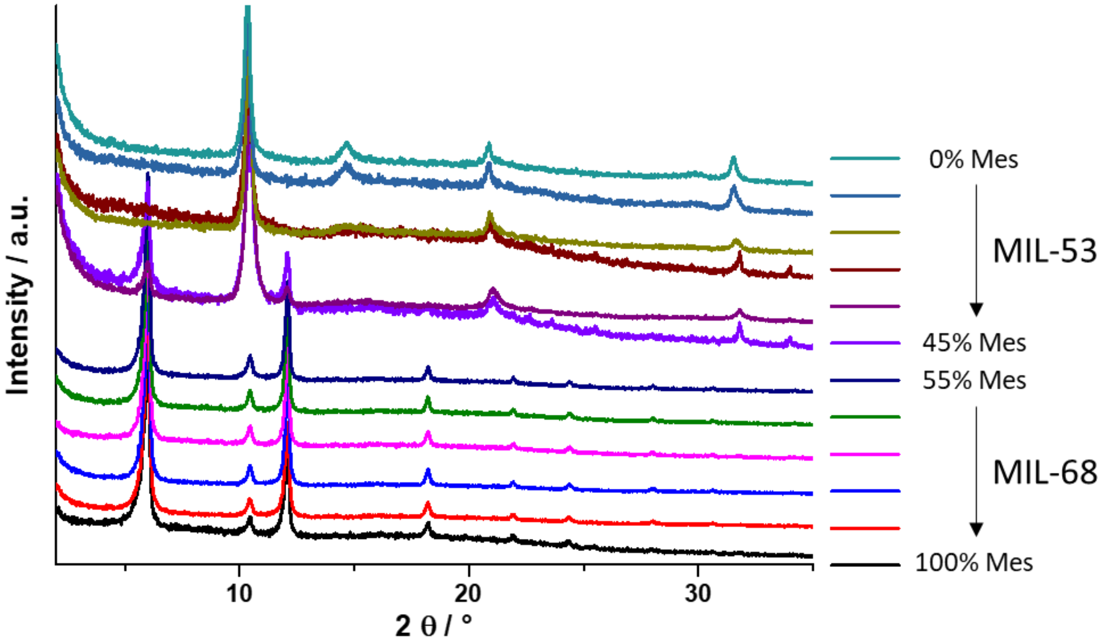

Figure S10.3 X-ray diffractograms of the mixed-linker fr-Al-MIL-53/68-Fum/Mes samples prepared in the flow reactor after activation and N_2 physisorption measurements. The crystal structure changes between 45% and 55% mesaconate content. No crystallinity loss is can be detected when compared to the pristine materials (Figure S2.3).

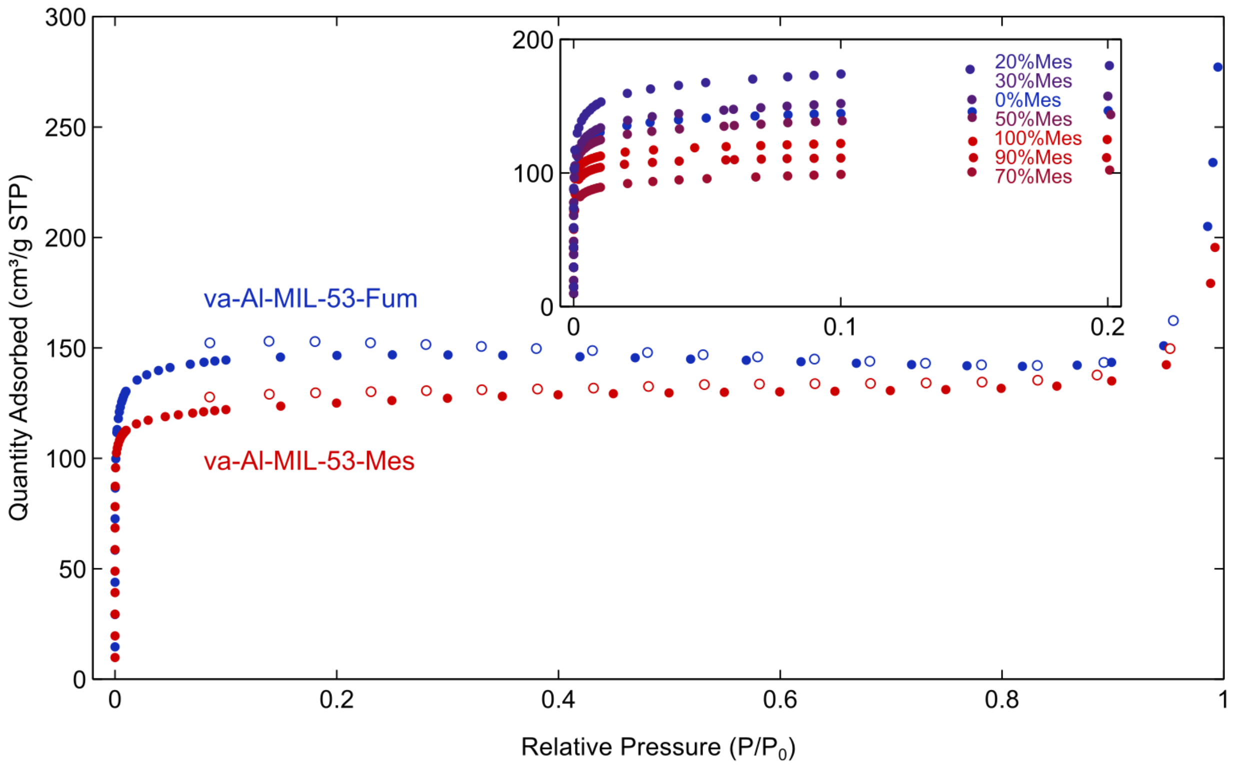

Figure S10.4 N_2 physisorption isotherms measured at -196 °C for the materials prepared via vapour-assisted synthesis. The main graph shows both the adsorption (full circles) as desorption (empty circles) isotherm branches of va-Al-MIL-53-Mes (blue) and va-Al-MIL-53-Fum (red). The inset shows a magnification of the adsorption branch in the microporous range for various mixed-linker products.

Table S10.2 Specific surface area and micropore volume extracted from N_2 physisorption data for the materials prepared via vapour-assisted synthesis.

Material	$a_{s,BET}$* / $m^2 \cdot g^{-1}$	$V_{mic.}$# / $cm^3 \cdot g^{-1}$
**va-Al-MIL-53-Mes (calc.)**	-	0.210
**va-Al-MIL-53-Mes**	527	0.169
**va-Al-MIL-53-90%Mes**	459	0.169
**va-Al-MIL-53-80%Mes**	-	-
**va-Al-MIL-53-70%Mes**	400	0.126
**va-Al-MIL-53-60%Mes**	-	-
**va-Al-MIL-53-50%Mes**	562	0.178
**va-Al-MIL-53-40%Mes**	-	-
**va-Al-MIL-53-30%Mes**	610	0.167
**va-Al-MIL-53-20%Mes**	703	0.218
**va-Al-MIL-53-10%Mes**	826	0.260
**va-Al-MIL-53-Fum**	592	0.205

*The specific surface area was calculated following the Rouquerol criteria.[7] #t-plot micropore volume, or theoretical value calculated using Platon[12] and a probe with a diameter 1.2 Å for the refined crystal structure protonated using Material Studio.[10]

S11. Vapour pressure determination of mesaconic acid and fumaric acid via thermogravimetry

The vapour pressure as a function of temperature was extracted from thermogravimetric measurement of the ligand powder loaded in a Knudsen cell. The thermogravimetric measurement was performed in vacuum under inert conditions and with temperature profile consisting of a series of isothermal steps (Figure S11.1). The mass loss rate as a function of temperature was calculated from the smoothened derivative of the mass loss in a steady portion of the corresponding isothermal step (green boxes, Figure S11.2). The vapour pressure as a function of temperature could then be calculated using the Knudsen equation (Fig. S11.3)[13]:

$$P = \frac{dm}{dt}\frac{\sqrt{\frac{2\pi RT}{M}}}{WA}\ [\mathrm{Pa}]$$ is the vapour pressure at temperature T [K]

where $$W = \frac{1}{(1+\frac{3l}{8r})}$$

$A = 2\pi r^2\ [\mathrm{m}^2]$ is the Knudsen cell orifice area

$l = 0.0003\ \mathrm{m}$ is the Knudsen cell orifice length

$r = 4.85 \times 10^{-5}\ \mathrm{m}$ is the Knudsen cell orifice radius

$M\ [\frac{\mathrm{g}}{\mathrm{mol}}]$ is the compound molar mass

$\frac{dm}{dt}\ [\frac{\mathrm{kg}}{\mathrm{s}}]$ is the mass loss rate at temperature T [K]

The vapour pressure as a function of temperature (Fig XY) could then be fitted to the integral form of the Clausius-Clapeyron equation, assuming a constant specific heat of evaporation L, ideal gas behavior for the vapour and a negligible molar volume of the solid phase, and with C a constant parameter (Figure S11.4):

$$\ln P = C + \frac{L}{RT}$$

The thermogravimetric study confirms the higher volatility of mesaconic acid at 80 °C (Table S11.1).

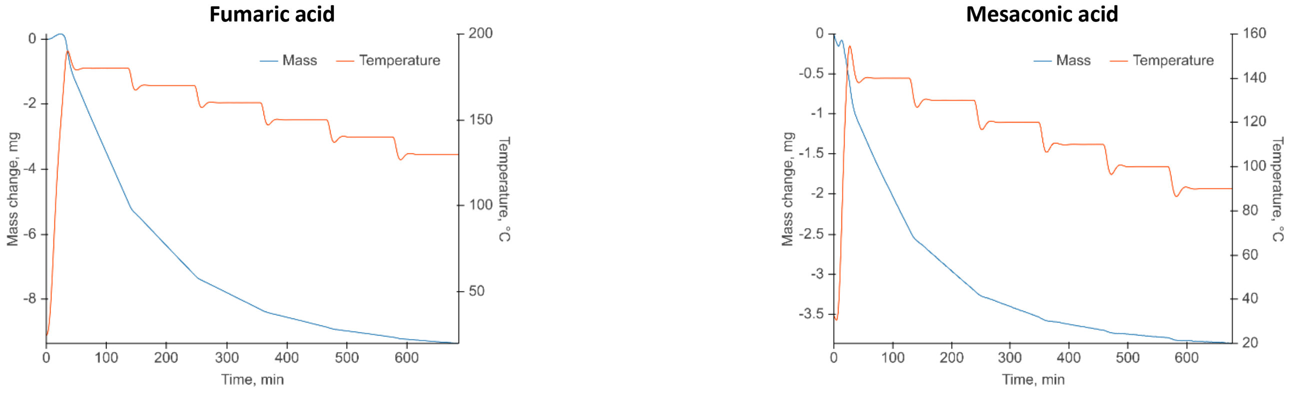

Figure S11.1 Thermogravimetric data for the vapour pressure determination of fumaric acid (left) and mesaconic acid (right).

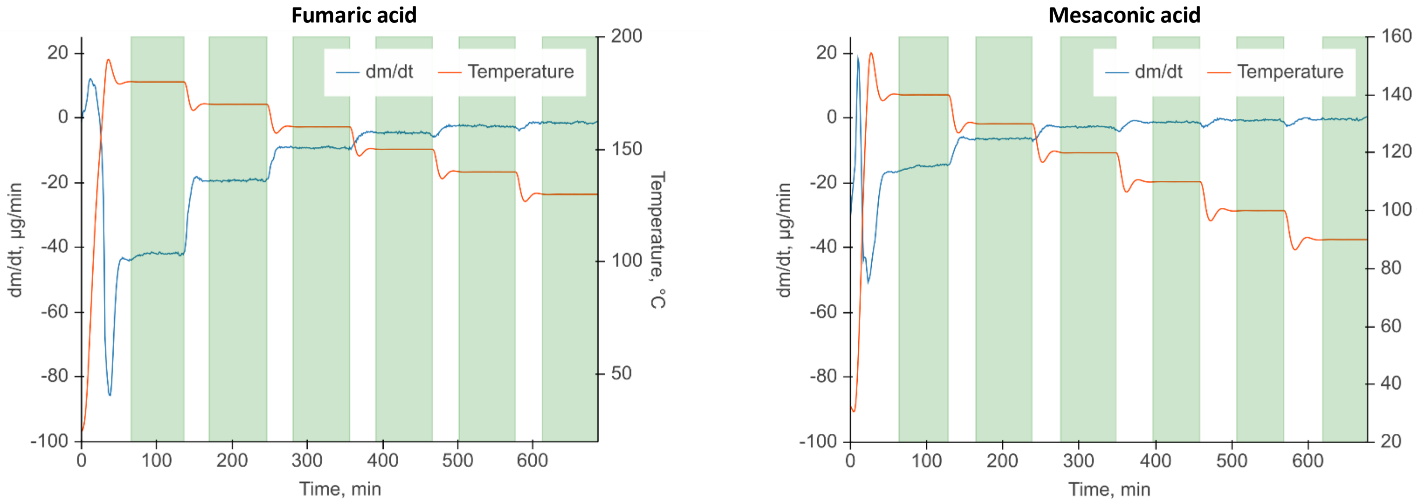

Figure S11.2 Mass loss rate from thermogravimetric data of fumaric acid (left) and mesaconic acid (right).

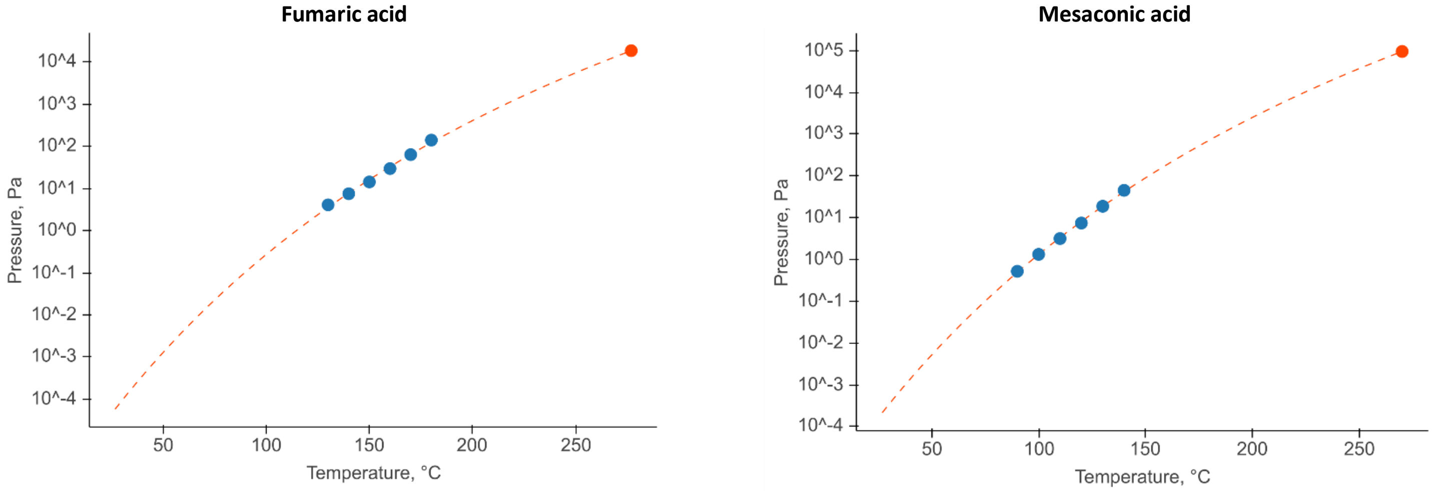

Figure S11.3 Vapour pressure (plain blue markers) as a function of temperature calculated with the Knudsen cell equation for fumaric acid (left) and mesaconic acid (right), and fitted Clausius-Clapeyron equation (dashed orange line) and predicted boiling point (i.e. temperature for P = 1 atm, orange marker).

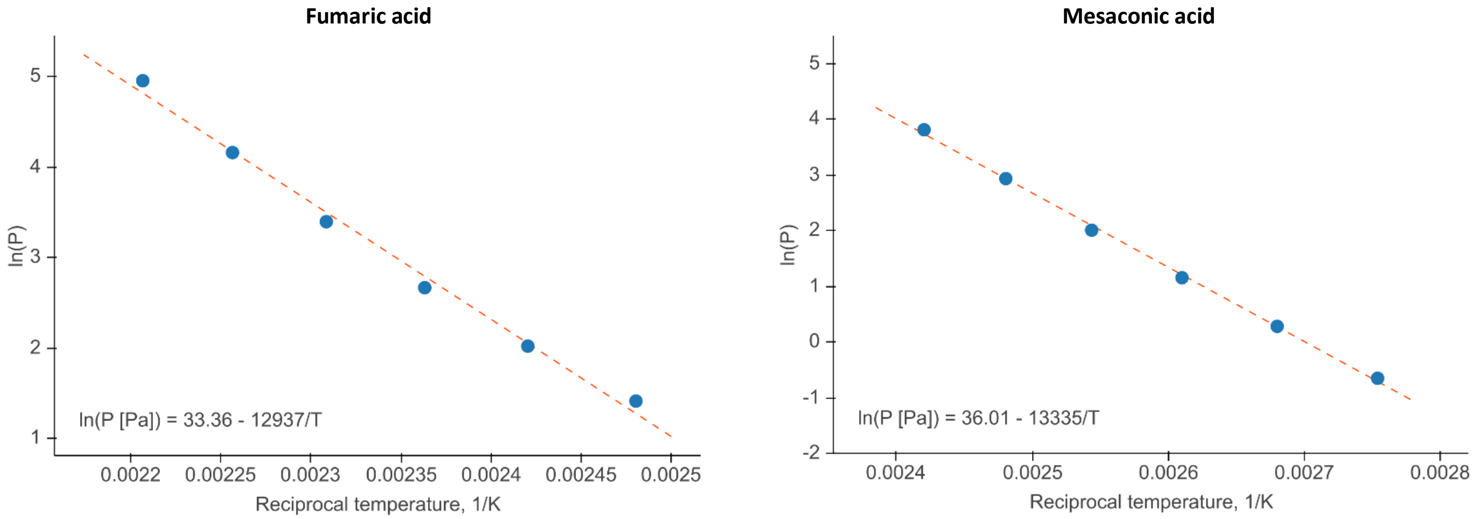

Figure S11.4 Linear fit to the Clausius-Clapeyron equation for fumaric acid (left) and mesaconic acid (right).

Table S11.1 Summary of the vapour pressure determination of fumaric acid and mesaconic acid via thermogravimetry.

	Fumaric acid	Mesaconic acid	Ref.
**Clausius-Clapeyron fit**	$\ln P\,[\mathrm{Pa}] = 33.4 - \frac{12937}{T\,[\mathrm{K}]}$	$\ln P\,[\mathrm{Pa}] = 36.1 - \frac{13335}{T\,[\mathrm{K}]}$	This work
**Fit R^2 value**	0.9926	0.9986	This work
**L – Latent heat of sublimation**	107.6 kJ/mol	110.9 kJ/mol	This work
**Vapour pressure at 80 °C**	0.039 Pa	0.171 Pa	This work
**Predicted Boiling point**	315 °C	270 °C	This work
**L – Latent heat of sublimation**	134.0 ± 4.2 kJ/mol for T = 85-98 °C	-	[14]
**Boiling point**	290 °C	250 °C (decomp.)	[14,15]

S12. Positronium Annihilation Lifetime Spectroscopy

Table S12.1. Results of PALS measurements performed on fr-Al-MIL-53-Fum (space group $P2_1/c$) and va-Al-MIL-53-Fum (space group *Pnma*).

**Al-MIL-53-Fum**	**Energy**	***o*-Ps1**			***o*-Ps2**			***o*-Ps3**		
**Space group**	**(keV)**	**Lifetime (ns)**	**Intensity (%)**	**d (Å)**	**Lifetime (ns)**	**Intensity (%)**	**Diameter (Å)**	**Lifetime (ns)**	**Intensity (%)**	**Diameter (Å)**
$P2_1/c$	**1.5**	2.11 ± 0.04	4.7427	5.90268	35.44 ± 0.59	6.7849	29.6902	/	/	/
Pnma	**1.5**	1.06 ± 0.04	11.4262	3.50914	4.51 ± 0.31	2.8174	9.01672	28.26 ± 1.59	4.3356	25.0606

S13. References

1 N. Heidenreich, U. Rütt, M. Köppen, A. K. Inge, S. Beier, A.-C. Dippel, R. Suren and N. Stock, *Rev. Sci. Instrum.*, 2017, **88**, 104102.
2 M. M. Eldrup, D. Lightbody and J. N. Sherwood, *Chem. Phys.*, 1981, **63**, 51–58.
3 S. J. Tao, *J. Chem. Phys.*, 1972, **56**, 5499–5510.
4 P. Sperr, W. Egger, G. Kögel, G. Dollinger, C. Hugenschmidt, R. Repper and C. Piochacz, *Appl. Surf. Sci.*, 2008, **255**, 35–38.
5 P. Kirkegaard, J. V. Olsen and M. M. Eldrup, *PALSfit3: A software package for analysing positron lifetime spectra A software package for analysing positron lifetime spectra*, 2017.
6 S. Waitschat, M. T. Wharmby and N. Stock, *Dalton Trans.*, 2015, **44**, 11235–11240.
7 J. Rouquerol, P. Llewellyn and F. Rouquerol, *Stud. Surf. Sci. Catal.*, 2007, 49–56.
8 E. Alvarez, N. Guillou, C. Martineau, B. Bueken, B. Van de Voorde, C. Le Guillouzer, P. Fabry, F. Nouar, F. Taulelle, D. De Vos, J.-S. Chang, K. H. Cho, N. Ramsahye, T. Devic, M. Daturi, G. Maurin and C. Serre, *Angew. Chem. Int. Ed.*, , DOI:10.1002/anie.201410459.
9 H. Reinsch and D. De Vos, *Microporous Mesoporous Mater.*, 2014, **200**, 311–316.
10 *Accelrys Materials Studio*, Accelrys, 2009.
11 *Topas Academics*, Coelho Software, 2007.
12 P. van der Sluis and A. L. Spek, *Acta Crystallogr. A*, 1990, **46**, 194–201.
13 M. A. V. R. Da Silva and M. J. S. Monte, *Thermochim. Acta*, 1990, **171**, 169–183.
14 P. Linstrom, 1997.
15 ChemicalBook Website, 2006.
